# Parameter Estimation of Microkinetic Models for Heterogeneous Catalysis

**DOI:** 10.1021/acs.iecr.6c01417

**Published:** 2026-07-15

**Authors:** M G Toufik Ahmed, Bimol Nath Roy, M. M. Faruque Hasan

**Affiliations:** † Artie McFerrin Department of Chemical Engineering, 14736 Texas A&M University, College Station, Texas 77843-3122, United States; ‡ Texas A&M Energy Institute, Texas A&M University, College Station, Texas 77843, United States

## Abstract

Microkinetic models (MKMs) provide insights and understanding of surface kinetics, reaction mechanisms, and catalyst behavior for complex reaction systems. Estimation of the MKM parameters can be posed as a nonlinear optimization problem (NLP). However, it is often difficult to solve the NLP due to model stiffness, ill-conditioning, and nonconvexity. In this work, we first formulate an NLP that allows the incorporation of experimental and computational data, DFT calculations, and semiempirical relations, to estimate the MKM parameters. We also propose a robust method with an adaptive feasibility restoration to solve the NLP. To address the ill-conditioning commonly encountered in catalytic reaction networks, the algorithm uses a minimum-norm least-squares search direction with a projected backtracking line search that enforces physical bounds without introducing additional inequality multipliers. We utilize the algorithm to estimate rate parameters for multitemperature experimental data across different reaction systems. The strategy improves numerical robustness, solution quality, and computational efficiency and provides a robust approach for microkinetic parameter estimation in heterogeneous catalysis. The MKM parameter estimation code is made available here: such as https://github.com/SOULS-TAMU/MKMParameterEstimation.git.

## Graphical abstract

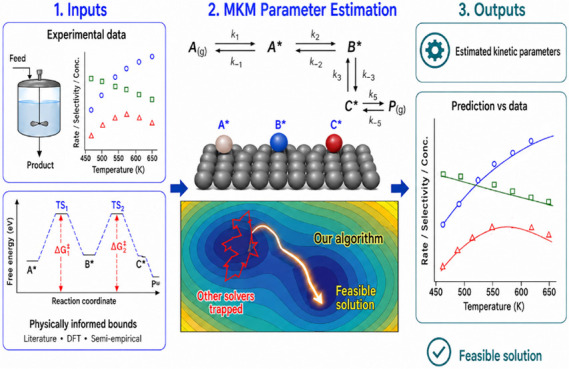

## 1 Introduction

Catalytic reactions are central to many industrial and environmental processes, offering high efficiency and selectivity through the use of active surfaces that lower activation barriers.^1^ Microkinetic models (MKMs) link atomistic-scale energy data with the microscopic and macroscopic behavior of a reaction system. MKMs avoid the assumptions regarding dominating surface species or reaction pathways by explicitly listing all elementary steps and surface intermediates.^2–4^ As a result, MKMs can predict reaction rates, dominant flux pathways, selectivity trends, surface coverages, and rate-determining steps directly from fundamental kinetic and thermodynamic inputs.^5–7^ To develop MKM for a heterogeneous catalytic process, a set of elementary reaction steps involving all gas-phase and surface-bound species is required. Kinetic parameters of these elementary reactions include activation energies and pre-exponential factors for rate expressions. Often, density functional theory (DFT) provides first-principles estimation of adsorption energies and transition-state barriers,^8–10^ Many MKMs have been developed based on solely DFT-based energy data as input.^8,11–15^ While DFT provides data on surface structures and reaction barriers, this alone is insufficient for capturing complex catalytic behavior.^10,16^ Traditional approaches often lack quantitative accuracy due to limitations in modeling adsorption/desorption energetics and coverage effects. Even high-level periodic DFT carries errors in activation energy from 9.6 to 20 kJ/mol, and for semiempirical relations (e.g., UBI-QEP,^17–19^ BEP^20,21^ ) this error is more than 20 kJ/mol.^3^ Such uncertainties can translate into significant discrepancies between simulated and experimental catalytic performance.^22^ Therefore, estimating kinetic parameters by fitting MKM to experimental data—rather than relying solely on *ab initio* values—has become a standard practice.^23,24^

When experimentally obtained conversion, product yields, or species concentration data are available, an optimization problem can be formulated to estimate microkinetic parameters by minimizing the errors between the estimated and experimentally observed data for a suitable reactor model.^25–28^ To optimize the MKM parameters, algorithms such as Nelder-Mead^29,30^ Rosenbrock^31^ Levenberg–Marquardt^23,32,33^ Bayesian optimization^34^ and genetic algorithms^35,37^ are often used. Many of these methods are limited by the number of problem size.^38,39^ Like other local optimization methods, Nelder-Mead’s performance strongly depends on the choice of initial guesses and may stagnate in nonconvex landscapes.^40^ The gradient-based Levenberg–Marquardt and Rosenbrock method are computationally expensive, sensitive to initial guesses, and often get trapped in local minima. Genetic algorithms, due to their stochastic nature, can escape local optima, but they demand extensive function evaluations. Furthermore, the complexity of the microkinetic and reactor models (high nonlinearity, stiffness, and large dimensionality) limits the generalizability of parameter estimation algorithms. As a result, the choice of algorithms and the quality of initial guesses significantly influence the accuracy and efficiency of MKM parameter estimation.^34^

Beyond parameter estimation, solving the MKM itself demands careful selection of solution strategies. Generally, steady-state output (concentration profile, mole fraction, selectivity, etc.) from the MKM is desired. This simplifies kinetic modeling and is effective for understanding catalyst behavior under fixed conditions.^41^ At steady-state, MKM yields a system of nonlinear algebraic equations whose solution corresponds to constant species concentrations and surface coverages. This can also be obtained through direct time integration of the full transient process until a steady-state solution is reached. Time integration is computationally expensive and requires stiff solvers like MATLAB’s ode23s. In contrast, the algebraic method, using solvers like MATLAB’s fsolve, is faster but notoriously sensitive to initial guesses. Poor guesses often lead to divergence or can be physically meaningless solutions.^42^ Another challenge in MKM parameter estimation arises from the inherent nonconvexity and degeneracy. They often lead to solution multiplicity, or the existence of multiple mathematically valid steady-state solutions for the same set of input conditions. As a consequence, solvers converge to local or even unstable steady states depending on the initial guess. Identifying the physically meaningful solutions requires additional stability analysis and expert knowledge from experimental trends, energy profiles from DFT or semiempirical relations, and deploying additional constraints.^1,3^ The multiplicity in solutions and degeneracy add significant complexity to the MKM workflow and must be carefully accounted for in model development.

To overcome these limitations, several approaches have been proposed in the past. Mundhwa et al.^29^ used a transient series CSTR model, which is equivalent to a 1-D plug flow reactor, to avoid nonphysically significant solutions when estimating MKM parameters.^43^ Although this ODE-based approach enables meaningful steady-state convergence, it requires a well-formed objective function. Constrained methods such as fmincon and lsqnonlin often fail to yield physically meaningful parameters, while the unconstrained fminsearch requires proper initialization and tuning.^29^ Several works addressed the parameter estimation problem as a dynamic problem and estimated MKM parameters by solving a system of ODEs.^23,29,36^

The central aim of this work is to develop an efficient and robust algorithm for estimating MKM parameters by solving steady-state algebraic equations arising from a defined network of elementary reaction steps. This problem arises in the context of heterogeneous catalysis, where accurate quantification of rate constants is essential for a mechanistic understanding and reactor-scale performance predictions. Specifically, we seek to determine the set of parameters that govern the rate expressions, namely the pre-exponential factors (A)/ sticking coefficient (s), temperature exponents (*b*), and activation energies (*E*_a_), of each elementary step, using data from multiple scales, which include observed concentration data measured across multiple experimental conditions, energetics data from DFT or other semiempirical relations, and any other operational/experimental/simulation data relevant to the system. We have formulated these different levels of prior information within a unified constrained NLP framework, where well-established quantities can be fixed, uncertain literature- or DFT-derived quantities can be imposed as physically informed bounds, and poorly characterized kinetic quantities can be treated as decision variables and fitted against the available data. This mixed-information formulation is then coupled to a solution strategy specifically designed for the ill-conditioned and rank-deficient systems that commonly arise in MKM parameter estimation.

Specifically, this work presents a nonlinear optimization (NLP)–based algorithm for developing MKM from steady-state reaction data obtained from multiple operating conditions. Several prior studies have also formulated MKM parameter estimation as a constrained NLP problem that may include thermodynamic consistency constraints.^44^ However, they often suffer from severe numerical difficulties due to the strong nonlinearity and ill-conditioning inherent in catalytic reaction networks. To improve numerical stability and optimization performance, we develop a Karush–Kuhn–Tucker (KKT)-based iterative algorithm. First, we present an updated NLP model that enables incorporation of information from heterogeneous data sources. In particular, the formulation allows simultaneous integration of process-level observations (e.g., concentration profiles, selectivity, and conversion) and molecular-scale information such as activation energies, pre-exponential factors, and temperature exponents obtained from DFT calculations. This capability is especially relevant in MKM studies, where some elementary reaction steps are well characterized by first-principles calculations, while others require calibration against experimental data. Second, we formulate and apply a strategy to solve the NLP-based MKM parameter estimation problem. Specifically, we obtain a system of equations that result from the KKT-based stationarity conditions of the NLP. We solve this system of equations using a Gauss–Newton scheme. The main contribution is the development of a KKT residual-correction algorithm that integrates minimum-norm pseudoinverse-based search directions, feasibility restoration, bound handling, and step-acceptance safeguards into a single solution framework. Although these individual components are well established in numerical optimization, their integration is designed specifically for ill-conditioned NLPs like MKM parameter estimation problems. The minimum-norm correction is used as the primary KKT search direction, rather than as a fallback, so that rank deficiency and underdetermination are handled directly during the iterative update. We address two closely related challenges here: (i) reliable estimation of kinetic parameters governing the elementary reactions, and (ii) robust computation of steady-state solutions of the resulting nonlinear algebraic system. The performance of the proposed algorithm is evaluated against several widely used commercial optimization solvers, including BARON^45^ IPOPT^46^ CONOPT^47^ and GUROBI^48^ thereby providing a systematic assessment of robustness and solution quality for complex MKM parameter estimation problems.

The remainder of this Article is organized as follows. Section 2 formulates the MKM parameter estimation problem and presents the corresponding NLP representation. Section 2 introduces the proposed solution strategy and describes the algorithms developed to solve the estimation problem. Section 4 provides a comparative analysis of the proposed algorithm against several conventional optimization solvers across several case studies. Finally, Section 5 summarizes the key findings and discusses potential directions for future research.

## 2 Microkinetic Modeling (MKM)

Microkinetic models inherently offer a multiscale perspective of chemical reaction systems, linking atomic and molecular phenomena with reactor performance. It integrates the microscopic details of adsorbate–catalyst interactions, the mesoscopic collective behavior of the reacting species, and the macroscopic realities of fluid mechanics and transport phenomena.^20^ The microkinetic model is constructed by listing the relevant elementary reaction steps in detail and determining individual rate parameters that represent the surface chemistry, such that the model predicts observable phenomena.

We consider a heterogeneous catalytic reaction network involving gaseous reactants and products, reaction intermediates, and vacant adsorption sites. Let I denote the complete set of chemical species involved in the system. This set is partitioned into three disjoint subsets: $I_{G}$ is the set of all gaseous species, $I_{I}$ is the set of intermediate/surface species, and $I_{S}$ is the set of vacant sites (here, we are considering one vacant site). Similarly, let R represents the set of all elementary reactions, and K denotes the set of all experimental conditions (e.g., temperature or inlet composition) considered. $R_{\mathrm{ads}}$ denotes the set of adsorption reactions and $R_{\mathrm{sur}}$ denotes the set of surface and desorption reactions. Let *i* and *j* denote chemical species and elementary reaction, respectively.

The goal is to find the MKM parameters to match experimental gas-phase concentrations across all k∈K . For each condition *k*, the model predicts the concentration $x_{i,k}$ of every species *i*, with experimental measurements available for all $i\in I_{G}^{\mathrm{data}}$ . Let, $I_{G}^{\mathrm{data}}$ represent the set of the gaseous species for which experimental data are available. These model-predicted values must satisfy mass balances derived from a steady-state continuous stirred-tank reactor (CSTR) assumption, in conjunction with thermodynamically consistent rate laws and surface site constraints. Let, $\left|I_{G}\right|$ represent the number of gas-phase species, $\left|I_{I}\right|$ represent the number of surface intermediates, and $\left|I_{S}\right|$ represent the number of site species (we are considering one). Then, $\left|I\right|=\left|I_{G}\right|+\left|I_{I}\right|+\left|I_{S}\right|$ , where |I| represent the total number of species in the system. Furthermore, $I=I_{G}\cup I_{I}\cup I_{S}$ .

The rate of reaction *j* is expressed using a generalized mass-action form:

1 $$r_{j}=k_{j}\underset{i\in I}{\prod }x_{i}^{n_{i,j}},,j\in R$$

where *x*_*i*_ denotes the concentration of species *i* (gas-phase concentration for gas species and surface coverage for surface species), and *n*_*i,j*_ represents the stoichiometry number of reactant *i* in reaction *j*. For the rate expression, we are considering each elementary step as irreversible. For adsorption of a gas species *i* onto vacant site (*), the rate expression assumes the following form:

2 $$r_{j}=k_{j}C_{i}\theta_{*},,j\in R_{\mathrm{ads}}$$

where *C*_*i*_ is the concentration of gas species *i* participating in reaction *j* and θ_*_ is the fractional coverage of vacant catalyst sites. The adsorption rate constant is calculated using collision theory:^1,2,49,50^

3 $$k_{j}=s_{j}{\left(\frac{RT}{2\pi M_{i,j}}\right)}^{1/2},,j\in R_{\mathrm{ads}}$$

where *s*_*j*_ is the sticking coefficient for the adsorption step *j*, *R* is the universal gas constant in (g·m^2^)/(s^2^·mol·K), *M*_*i,j*_ is the molecular weight of gas species *i* in reaction *j* in g/mol, and *T* is the temperature in K. For desorption and surface reactions, the rate constant follows an modified Arrhenius-type expression based on transition state theory:^50–53^

4 $$k_{j}=A_{j}{\left(\frac{T}{T_{ref}}\right)}^{b_{j}}\exp\left(-\frac{Ea_{j}}{RT}\right)S_{den},,j\in R_{\mathrm{des}}\cup R_{\mathrm{sur}}$$

where *A*_*j*_ (1/s) and *E*_*a*_ (kJ/mol) are the pre-exponential factor and activation energy of reaction *j*, respectively. *S*_*den*_ is site density which has a unit of mol/*m* ^2^ and it is multiplied with the rate constant to maintain dimensional homogeneity. *T*_*ref*_ is the reference temperature, and *b* is the temperature exponent. Here, the activation energy is treated as independent of surface coverage. However, in systems with strong adsorbate interactions or coverage-dependent transition states, the activation barrier may depend on the surface coverage and can be posed as a nonlinear function of surface coverage in the rate constant expression.

In this work, we assume that all the reactions occur within a CSTR. The gas-phase species conservation at steady state in CSTR is given by

5 $$\frac{dx_{i}}{dt}=0=\frac{x_{i}^{\mathrm{in}}-x_{i}}{\tau }+\frac{A^{sur}}{V}\underset{j\in R}{\sum }\varepsilon_{i,j}\nu_{i,j}r_{j},,i\in I_{G}$$

Here, *x*_*i*_ denotes the outlet concentration of gas-phase species $i\in I_{G}$ , and $x_{i}^{0}$ refers to the inlet concentration of species *i*. τ, and *A* ^*sur*^/*V*, represent the space time, catalyst surface area-to-volume ratio, respectively. We assume that these parameters are known. In case they are often not readily available in the literature, τ, and *A* ^*sur*^/*V*, may be used as tuning parameters.^24^

The conservation of surface species at steady state is given by

6 $$\frac{dx_{i}}{dt}*S_{den}=0=\underset{j\in R}{\sum }\varepsilon_{i,j}\nu_{i,j}r_{j},,i\in I_{I}$$

7 $$\underset{i\in I_{S}\cup I_{I}}{\sum }x_{i}=1$$

Here, *x*_*i*_ denotes fractional surface coverage of surface species $i\in I_{I}$ .

We would like to estimate the full vector of kinetic parameters $\pi ={\left[A_{j},s_{j},b_{j},Ea_{j}\right]}^{T}$ for all j∈R in a manner that minimizes the model’s error across all operating conditions. The following NLP formulation enables a mechanistically sound and data-driven calibration of MKMs, laying the groundwork for further mechanistic analysis, sensitivity studies, and process-scale reactor simulations. The estimation of kinetic parameters is posed as an optimization problem. The objective function minimizes the squared error between experimental and model-predicted gas-phase concentrations, aggregated across all conditions. The optimization is subject to a set of equality constraints that include species mass balances, and rate expressions for each elementary reaction. Additional inequality constraints enforce physical bounds on species concentrations (e.g., 0 ≤ *x*_*i,k*_ ≤ 1). Each reaction rate is expressed using a modified Arrhenius form, incorporating pre-exponential factors, temperature exponents, and activation energies, all of which are to be estimated from data.

Let $x_{i,k}^{\mathrm{data}}$ denote the experimentally observed concentration of gas species *i* under condition *k*, and $x_{i,k}^{\mathrm{in}}$ the corresponding inlet concentration. The rate of reaction *j* under condition *k* is represented by $r_{j,k}$ . Each species’ role in the reactions is defined using the rate direction indicator *ε*_*i,j*_ and the stoichiometric coefficient ν_*i,j*_, while *n*_*i,j*_ represents kinetic exponents of component *i* in reaction *j*. Temperature dependence of reaction rates is captured through the modified Arrhenius equations. For each reaction *j*, the rate constant *k*_*j*_ is defined by a pre-exponential factor *A*_*j*_, temperature exponent *b*_*j*_, and activation energy *E*_*j*_, all of which are parameters to be estimated. These parameters vary across reactions and govern the temperature sensitivity of the elementary step kinetics. The parameters and variables are summarized in Table 1.

**Table 1:** Summary of Key Parameters and Variables

Symbol	Description
Parameters	
$$x_{i,k}^{\mathrm{data}}$$	Experimentally measured concentration of species *i* at *k*, for $i\in I_{G}$ (mol/m^3^)
$x_{i,k}^{\mathrm{in}}$	Inlet concentration of gas-phase species *i* under condition *k* (mol/m^3^)
*ε*_*i,j*_	Rate direction indicator for species *i* in reaction *j* (−), takes a value of −1 or 1 for reactant or product, respectively
ν_*i,j*_	Stoichiometric coefficient for species *i* in reaction *j* (−)
*n*_*i,j*_	Kinetic exponent of the reactant species *i* in the reaction of step *j* (−)
*T̂*_*k*_	Known temperature corresponding to experimental condition *k* (K)
τ	Reactor residence time (s)
*A* ^*sur*^/*V*	Area-to-volume ratio of the catalyst (1/m)
*S*_*den*_	catalyst site density (*mol*/*m* ^2^)
*R*	Universal gas constant (8.314 J/mol·K)
Intermediate variables	
*x*_*i,k*_	Concentration of species *i* under condition *k* (mol/m^3^/-)
*r*_*j,k*_	Net rate of elementary reaction *j* at condition *k* (−)
*k*_*j,k*_	Rate constant of elementary step *j* at condition *k* (1/s)
Decision variables	
*A*_*j*_	Pre-exponential factor (1/s), for $j\in R_{\mathrm{sur}}$
*s*_*j*_	Sticking coefficient (−), for $j\in R_{\mathrm{ads}}$
*b*_*j*_	Temperature coefficient (−), for $j\in R_{\mathrm{sur}}$
*Ea*_*j*_	Activation energy (J/mol), for $j\in R_{\mathrm{sur}}$

With these, the complete NLP model formulation *P* is as follows:

8a $$P:\underset{\pi }{\min}\underset{i\in I_{G}^{\mathrm{data}}}{\sum }\underset{k\in K}{\sum }{\left(x_{i,k}^{\mathrm{data}}-x_{i,k}\right)}^{2}$$

8b $$\mathrm{s.t.}\frac{x_{i,k}^{\mathrm{in}}-x_{i,k}}{\tau }+\frac{A}{V}\underset{j\in R}{\sum }\varepsilon_{i,j}\nu_{i,j}r_{j,k}=0,,i\in I_{G},k\in K$$

8c $$\underset{j\in R}{\sum }\varepsilon_{i,j}\nu_{i,j}r_{j,k}=0,,i\in I_{I},k\in K$$

8d $$\underset{i\in I_{S}\cup I_{I}}{\sum }x_{i,k}=1,,k\in K$$

8e $$r_{j,k}=k_{j,k}\underset{i\in I}{\prod }x_{i,k}^{n_{i,j}},,j\in R,k\in K$$

8f $$k_{j,k}=s_{j}{\left(\frac{R{\mathrm{T̂}}_{k}}{2\pi M_{i}}\right)}^{1/2},,j\in R_{\mathrm{ads}},k\in K$$

$$k_{j,k}=A_{j}{\mathrm{T̂}}_{k}^{b_{j}}\exp\left(-\frac{{Ea}_{j}}{R{\mathrm{T̂}}_{k}}\right)*S_{den},,j\in R_{\mathrm{sur}},k\in K$$ 8g

8h $$0\leq x_{i,k}\leq 1,,i\in I_{S}\cup I_{I},k\in K$$

8i $$0\leq x_{i,k},,i\in I_{G},k\in K$$

Eqs 8–16 are used for the MKM case studies described in Section 4.4. Eq 8 represents the objective function that minimizes the squared error between experimental and model-predicted concentrations of gaseous species. The optimization is subject to a set of physical and chemical constraints. We assume the reactor operates under steady-state with constant flow, allowing us to apply mole balances for gaseous species, given by eq 9, where τ is the residence time, and (*A* ^*sur*^/*V*) is the catalyst surface area per unit reactor volume. The coverage of intermediate surface species must also satisfy steady-state conditions. These balances are expressed in eq 10. Additionally, total surface coverage is conserved through eq 11. Eq 12 represents the net rate for each elementary reaction through the generalized mass-action law. The exponents *n*_*i,j*_ is derived from the absolute stoichiometric matrix ν_*i,j*_, based on if species *i* is a reactant in reaction *j*.

9 $$n_{i,j}=\left\{ \begin{array}{ll} \nu_{i,j}, & \mathrm{if}\;\varepsilon_{i,j}<0\\ 0, & \mathrm{otherwise} \end{array}\right.$$

Eqs 13–14 define the rate constants. The model imposes physically meaningful bounds on species concentrations, as described by eqs 15–16, where, as the surface intermediates are expressed as fractions with values between 0 and 1. For gaseous species, concentration can only be positive, implying the lower bound being zero. An illustrative example is provided in the Supporting Information. Note that, model *P* does not include any bounds on the decision variables *A*_*j*_, *b*_*j*_, *Ea*_*j*_. However, additional constraints for bounding these variables can be obtained from insights, experiments, through molecular/atomic simulations like DFT, or semiempirical relations and can be added in the following form of inequality constraints.

10a $${\left[A_{j}\right]}^{L}\leq A_{j}\leq {\left[A_{j}\right]}^{U},,j\in R$$

10b $${\left[b_{j}\right]}^{L}\leq b_{j}\leq {\left[b_{j}\right]}^{U},,j\in R$$

10c $${\left[Ea_{j}\right]}^{L}\leq Ea_{j}\leq {\left[Ea_{j}\right]}^{U},,j\in R$$

where superscript L denotes the lower bound and superscript U denotes the upper bound for the corresponding variable. These additional constraints are added on the basis of the availability of system knowledge.

## 3 Solution Strategy

Initial attempts to solve the NLP with commercial solvers showed inconsistent performance across the tested cases. Although a small subset of instances converged to feasible solutions, most runs failed to identify a reliable feasible region. In representative runs, BARON reported infeasibility without returning a solution, IPOPT converged to a locally infeasible point after a large number of iterations, and CONOPT terminated at an intermediate infeasible point with an additional warning of discontinuous derivative behavior. The profound impact of scaling is evident in several instances where the BARON solver’s output shifted from infeasible to feasible strictly following the appropriate scaling of the pre-exponential factors, activation energies, and governing equations. However, inspection of the solver logs suggests that these failures cannot be attributed to scaling alone. A plausible explanation is the combined effect of highly nonlinear kinetic expressions, strong correlation among kinetic parameters, active parameter bounds, and persistent violations in the coupled gas-phase balance equations. This is strongly nonconvex constrained system, in which the feasible region is narrow and difficult to locate consistently. These characteristics induce strong parameter dependencies and reduce the effective rank of the Jacobian, resulting in an ill-conditioned or near-singular system. Consequently, the problem becomes highly sensitive to perturbations, and the search directions computed by standard NLP solvers become unreliable. For interior-point methods such as IPOPT, sensitivity to the initial point and convergence to locally infeasible stationary points are well-documented behaviors in nonconvex nonlinear programming problems.^46,54^ Similarly, solvers such as CONOPT rely on smooth and well-defined derivatives, and their performance can deteriorate in the presence of discontinuities or irregular gradient information.^55^ Global optimization solvers such as BARON may report infeasibility or fail to identify feasible solutions when the feasible region is difficult to locate within the search space.^56^ As a result, while commercial solvers may succeed in some instances, they do not provide robust and reliable convergence across the full set of cases considered. This motivates the development of a solution strategy that stabilizes the search direction and handles near-singular Jacobians more effectively.

The governing microkinetic equations are formed as equality constraints, while physical bounds on the variables are handled through projection. To estimate the optimal kinetic parameters and enforce the governing microkinetic equations, we frame the parameter estimation as a constrained NLP problem. Let $v={\left[x^{T},p^{T},r^{T}\right]}^{T}\in R^{n}$ , with **l** ≤ **v** ≤ **u**, represent the concatenated vector of primal variables. Here, **x** denotes species concentrations and surface coverages, **p** represents kinetic parameters, and **r** corresponds to the rates of elementary reactions. Initial values of the reaction rates are computed consistently from the kinetic expressions using the initial guesses of **x** and **p**. Experimental measurements are available only for a subset of observable states **x**_*obs*_. The NLP model is formulated as

11 $$\underset{x,p}{\min}F\left(x_{obs}\right)={∥x_{obs}^{data}-x_{obs}∥}_{2}^{2}$$

12 s.t.h(x,p,r)=0

where **h**(·) represents the steady-state algebraic system of equations derived from the reaction network. Both the model states and kinetic parameters are treated as optimization variables, allowing the microkinetic equations to be enforced directly as equality constraints. Eq 8–16 formulate the MKM parameter estimation problem for a steady-state, gas-phase CSTR. Later, eqs 21–22 provide the same formulation in a general form.

This equality-constrained problem is handled using the Lagrangian:

13 $$L\left(v,\lambda \right)=F\left(x_{obs}\right)+\lambda^{T}h\left(v\right)$$

where **λ** are the Lagrange multipliers associated with the model equations. The Karush–Kuhn–Tucker (KKT) equations, which describe the first-order stationary conditions for the solution of the NLP problem, require that both the gradient of the Lagrangian and the constraints evaluate to zero. Defining the full variable vector **z** = [**v** ^T^, **λ** ^T^]^T^ the KKT residual vector is defined as

14 $$r\left(z\right)=\left[\begin{array}{c} \nabla_{v}L\left(z\right)\\ h\left(v\right) \end{array}\right]$$

The objective of the algorithm is to solve this nonlinear system to obtain an optimal point z* such that **r**(z*) = 0. To iteratively locate z*, the nonlinear KKT residual function is linearized around the current iterate **z**_*k*_ using a first-order Taylor expansion:

15 $$r\left(z_{k}+\Delta z\right)\approx r_{k}+J_{k}\Delta z$$

where **r**_*k*_ = **r**(**z**_*k*_) and $J_{k}={\frac{\partial r}{\partial z}|}_{z_{k}}$ is the Jacobian of the residual system. The Jacobian possesses the following block structure:

16 $$J\left(z\right)=\left[\begin{array}{cc} \nabla_{v}^{2}L & \nabla_{v}h^{T}\\ \nabla_{v}h & 0 \end{array}\right]$$

where ∇_**v**_ **h** is the constraint Jacobian. In the present implementation, the Jacobian required in the KKT update is computed symbolically. For larger MKM networks, automatic differentiation tools (e.g., CASADI^57^ ADiMat^58^ etc), may offer a faster and more scalable way to compute derivatives. Enforcing the condition that the linear approximation equals zero yields the linear system:

17 $$J_{k}\Delta z=-r_{k}$$

The NLP formulation is inherently underdetermined due to parameter degeneracy and the limited information content typically found in experimental data sets. Kinetic parameters are often strongly correlated, allowing multiple parameter combinations to produce identical reaction rates. This leads to nonunique solutions and inherent rank deficiency in the Jacobian matrix **J**_*k*_.^59,60^ This structural underdetermination becomes increasingly severe when the number of kinetic parameters significantly exceeds the independent experimental observations.^61^ Consequently, the linear system **J**_*k*_ Δ ***z*** = -**r**_*k*_ exhibits degeneracy, possessing infinitely many valid search directions Δ**z** that yield comparable residuals. To resolve this, we first define the set of all least-squares solutions S that minimize the residual error:

18 $$S=\arg\underset{\Delta z}{\min}∥J_{k}\Delta z+r_{k}{∥}_{2}^{2}$$

From this set of degenerate solutions, we then select the unique step that minimizes the *L*_2_ norm of the parameter update:

19 $$\underset{\Delta z\in S}{\min}∥\Delta z{∥}_{2}$$

This two-step formulation ensures that among all vectors Δ**z** that minimize the residual, the solver selects the one requiring the smallest step in the parameter space. The unique solution^62,63^ to this problem is given by

20 $$\Delta z=-J_{k}^{†}r_{k}$$

where $J_{k}^{†}$ is the Moore-Penrose pseudoinverse.^64,65^ To compute $J_{k}^{†}$ robustly, we utilize the Singular Value Decomposition (SVD) of the Jacobian, $J_{k}=U\Sigma V^{T}$ .^62,66,67^ The pseudoinverse is constructed as $J_{k}^{†}=V\Sigma^{†}U^{T}$ , where **Σ** ^†^ is obtained by taking the reciprocal of singular values and setting any singular value below a threshold (ϵ_*pseudoinverse*_) strictly to zero.

Solving the linearized KKT system via pseudoinversion introduces two distinct forms of mathematical approximation. The first approximation arises from neglecting higher-order terms in the Taylor series expansion. This linearization error is of second order which means that the calculated step Δ**z** is only a valid descent direction within a local neighborhood of **z**_*k*_. To guarantee convergence despite this approximation, the method employs an adaptive line search strategy.^54^ By enforcing the Armijo sufficient-decrease condition^68,69^ the solver systematically reduces the step length α ∈ (0,1] until the linear model accurately reflects the local nonlinear landscape.

The second source of approximation arises from the SVD. In practice, near-zero singular values are truncated, which effectively removes components associated with directions of low sensitivity in the parameter space. Although this may be interpreted as a loss of information, these directions are typically poorly identifiable and dominated by numerical noise rather than experimental data in rank-deficient systems. Retaining such components would lead to ill-conditioned updates, as the inversion of small singular values amplifies numerical errors. Consequently, the exclusion of these near-zero values acts as an implicit spectral regularization mechanism, ensuring that the computed search direction Δ**z** remains stable, well-conditioned, and physically meaningful.^70,71^ Although this may reduce the local convergence speed, it prevents amplification of numerical noise and avoids excessively large updates along poorly identifiable parameter directions. The combination of adaptive line search and SVD regularization ensures a monotonic decrease in the KKT residual, guiding the solver toward a feasible least-squares solution.

Once an appropriate step size α is determined, the trial iterate for the primal variables is updated:

21 $$ṽ=v_{k}+\alpha \Delta v$$

Instead of introducing inequality multipliers to handle variable bounds, physical constraints are strictly enforced using a projection operator P(·) :

$$P\left(v_{i}\right)=\left\{ \begin{array}{ll} l_{i}, & v_{i}<l_{i}\\ v_{i}, & l_{i}\leq v_{i}\leq u_{i}\\ u_{i}, & v_{i}>u_{i} \end{array}\right.$$ 22

The updated primal variables are then computed as $v_{k+1}=P\left(ṽ\right)$ , ensuring all iterates remain strictly physically feasible. The dual variables **λ** are updated without projection. For general nonlinear inequality constraints, the present framework handles them by converting the inequalities into equality constraints through a squared-slack formulation. A nonlinear inequality of the form *g*(*v*) ≥ 0 can be converted into an equality constraint by introducing a continuous slack variable s, giving *g*(*v*) - *s* ^2^ = 0. The resulting equality can then be appended to the constraint vector *h*(*v*) = 0 and solved within the KKT residual-correction framework. The projection operator P(·) is utilized to enforce simple variable bounds, such as physical limits on fractional surface coverages (e.g., 0 ≤ *x*_*i,k*_ ≤ 1) and user-defined bounds on the kinetic parameter search space.

The algorithm employs several numerical parameters to control globalization and robustness. The key algorithmic parameters include the KKT residual tolerance ϵ_kkt_, the pseudoinverse cutoff ϵ_pseudoinverse_, and the backtracking factor β_backtrack_. A complete list of algorithmic parameters and their definitions is provided in Table S1 of the Supporting Information. A feasibility restoration phase is applied when the equality constraint violations become large during the iterative search. In this phase, only the state variables **x** are updated, while the kinetic parameters **p** remain fixed. The restoration step is computed by solving a least-squares problem based on the state Jacobian **J**_*h,x*_ = ∇_**x**_ **h**(**x**, **p**, **r**), yielding

23 $$\Delta x=-J_{h,x}^{†}h$$

where $J_{h,x}^{†}$ denotes the Moore–Penrose pseudoinverse. The updated state is then projected onto the feasible bounds. This state-only restoration step reduces constraint violations without perturbing the kinetic parameters. As a result, it prevents large infeasibility. The backtracking parameter β_*backtrack*_ ∈ (0,1) controls the reduction of the step size during the line search, while α_*min*_ defines the minimum allowable step length. The damping parameter δ_*damp*_ ∈ (0,1) is used to weaken the Lagrange multipliers when stagnation occurs. A stagnation counter *C*_*stag*_ is used to detect lack of residual reduction across successive iterations. The parameter ϵ_*stag*_ denotes a small tolerance used to detect stagnation in the residual reduction. The parameter *c*_1_ ∈ (0,1) controls the sufficient decrease condition in the line search. The singular value cutoff parameter ϵ_*pseudoinverse*_ serves as the primary spectral regularization technique to stabilize the pseudoinverse computation.
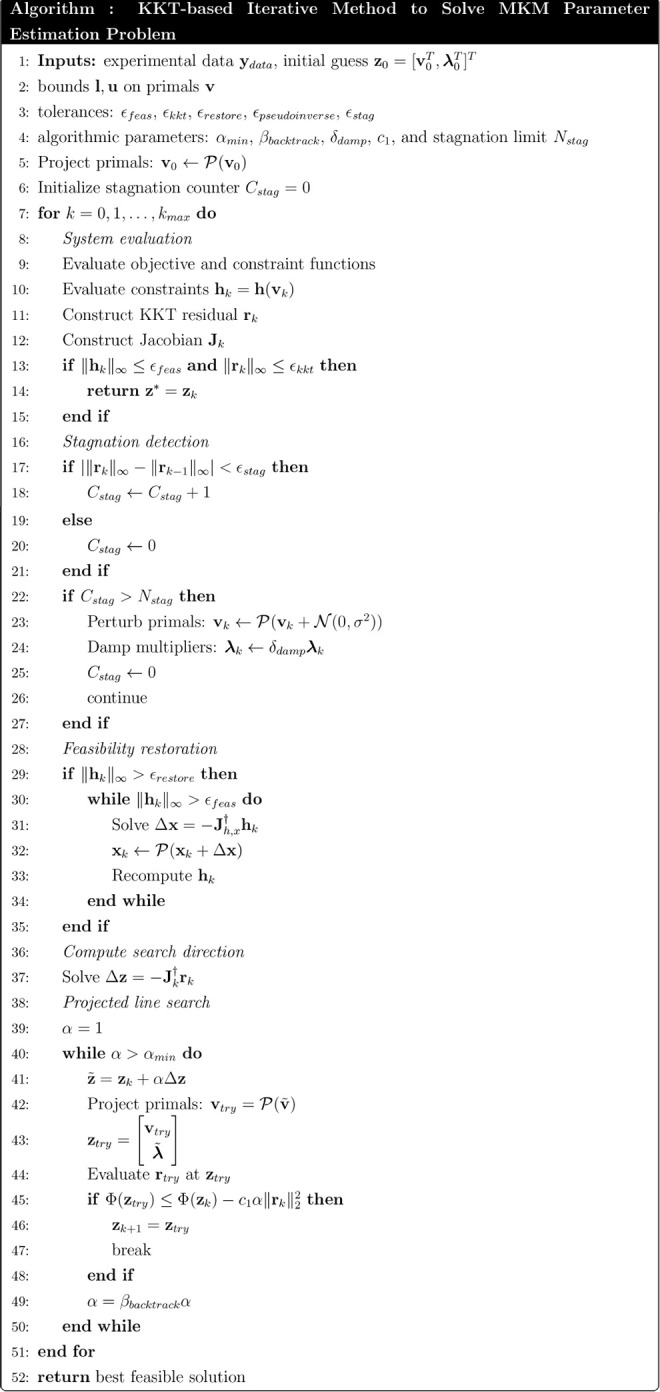


## 4 Results and Discussion

At first, we start with simple numerical experiments to check the algorithm’s efficiency. The first two examples (Section 4.1 and 4.2) serve as validation problems designed to verify that the proposed optimization method can correctly recover kinetic parameters from steady-state data. These problems involve a small number of unknown parameters and are relatively well-conditioned, allowing direct comparison with standard nonlinear programming solvers. The third case study (Section 4.3) introduces a more complex reaction network and provides a comparative analysis among the different solvers. Subsequently, we consider an MKM example to demonstrate how incorporating physically informed information about kinetic parameters can reduce solution multiplicity in Section 4.4. We have also presented three MKM case studies based on real experimental data to evaluate the performance of the proposed algorithm in practical catalytic systems. All numerical experiments were performed on a 13th Gen Intel Core i7–13700 (24-core CPU) with 32 GB RAM running Linux OS. Our algorithm and FMINSEARCH were run in MATLAB online^72^ where as the other solvers utilize GAMS 49.2.^73^ The case studies in this section contain nonlinear equality constraints from reactor balances and rate expressions, along with simple bound constraints on variables and kinetic parameters. Therefore, projection is used for bound handling in the present implementation. Although the present case studies focus on gas-phase heterogeneous catalysis, the NLP formulation can be (“conceptually”) extended to liquid-phase or aqueous systems by replacing the mass-action concentration terms with activity-based expressions and including the relevant phase-equilibrium or nonideal thermodynamic relations. However, one must be cognizant about the challenges that may arise from the generalized formulation.

### 4.1 Single Parameter CSTR

The first case study^74^ involves the liquid-phase hydrolysis of acetic anhydride to form acetic acid in a 1250-L isothermal CSTR. The overall reaction is given by

$$(CH_{3}CO{)}_{2}O+H_{2}O⇌2CH_{3}COOH$$

The reaction is first-order with respect to both acetic anhydride and water. The mass balance is shown below:

24 $$\frac{dx_{1}}{dt}=\frac{x_{1}^{\mathrm{in}}-x_{1}}{\tau }-\frac{A}{V}Kx_{1}x_{2}$$

25 $$\frac{dx_{2}}{dt}=\frac{x_{2}^{\mathrm{in}}-x_{2}}{\tau }-\frac{A}{V}Kx_{1}x_{2}$$

For this single-parameter CSTR example, eqs 34–35 are used in the parameter estimation problem by imposing their steady-state condition, *dx*_1_/*dt* = 0 and *dx*_2_/*dt* = 0. The resulting algebraic equations define the constraint vector *h*(*x*,*p*,*r*) = 0 in eq 22, with *K* treated as the unknown kinetic parameter. The corresponding dynamic CSTR model was integrated in MATLAB using the values provided in Table 2 to generate synthetic steady-state data for this validation case. They serve as the synthesized data for our first case study. The molar flow rates we obtained at steady state were put in the objective function, and we have tried to estimate *K*. The findings are provided in the Table 3.

**Table 2:** Model Parameters Used in the CSTR Example

$x_{1}^{\mathrm{in}}$ (mol/L)	$x_{2}^{\mathrm{in}}$ (mol/L)	*A* ^*sur*^/*V* (L)	τ (s/L)	*K* (L/(mol.s))
2.5	50	1250	1/15	0.0075

**Table 3:** Comparison of Parameter Estimation Results Across Different Solvers

Parameter	Ground Truth	Our Work	IPOPT	CONOPT	BARON	GUROBI
*K*	0.0075	0.00671	0.00671	0.00671	0.00671	0.00671
Time (s)	–	0.330	0.241	0.092	0.539	0.163
Objective Value	–	0.0001805	0.0001805	0.0001805	0.0001805	0.0001805

All solvers converge to essentially the same objective value (1.805 × 10^–4^) and parameter estimation indicates consistent accuracy across methods. However, computational time varies significantly, with CONOPT being the fastest (0.092 s) and BARON the slowest (0.539 s).

### 4.2 Simple Reaction Network

To illustrate the proposed parameter estimation algorithm, we consider a simple reaction network inspired by reaction schemes commonly used in chemical reaction engineering texts.^75^ The system consists of two parallel reactions:

2A→B,⁣A→C

The reactor is assumed to be a CSTR. Let *x*_1_, *x*_2_, and *x*_3_ denote the concentrations of species *A*, *B*, and *C*, respectively. The inlet concentrations are given as $x_{1}^{in}=30$ , $x_{1}^{in}=2$ and $x_{1}^{in}=1$ . The reactions follow mass action kinetics with rate constants *k*_1_ and *k*_2_. The reaction rates are

26 $$r_{1}=k_{1}x_{1}^{2},,r_{2}=k_{2}x_{1}$$

The dynamic mass balances for the reactor are written as

27 $$\frac{dx_{1}}{dt}=\left(30-x_{1}\right)+10\left(-k_{1}x_{1}^{2}-k_{2}x_{1}\right)$$

28 $$\frac{dx_{2}}{dt}=\left(2-x_{2}\right)+10\left(k_{1}x_{1}^{2}\right)$$

29 $$\frac{dx_{3}}{dt}=\left(1-x_{3}\right)+10\left(k_{2}x_{1}\right)$$

For this simple reaction-network example, eqs 36–39 define the reactor model. In the NLP formulation, the dynamic balances in eqs 37–39 are enforced at steady state by setting *dx*_1_/*dt* = 0, *dx*_2_/*dt* = 0, *dx*_3_/*dt* = 0. Together with the rate expressions in eq 36, these steady-state algebraic equations define the constraint vector *h*(*x*,*p*,*r*) = 0 in eq 22, where *p* = [*k*_1_,*k*_2_]^*T*^. Steady-state solutions are obtained when the concentration profiles are not changing anymore. To generate synthetic data, the system is first solved using the ground-truth parameters *k*_1_ = 0.5 and *k*_2_ = 1. The resulting steady-state concentrations are then used as target values in a nonlinear programming (NLP) formulation to estimate the unknown parameters *k*_1_ and *k*_2_. The findings are provided in the Table 4.

**Table 4:** Comparison of Parameter Estimation Results for the Toy Reaction Network Using Different Optimization Solvers

Parameter	Ground Truth	Our Work	IPOPT	CONOPT	BARON	GUROBI
*k*_1_	0.5	0.5	0.5	0.5	0.5	0.5
*k*_2_	1	1	1	1	1	1
Time (s)	–	0.379	0.12	0.119	0.628	0.122
Objective Value	–	2.70 × 10^–7^	2.70 × 10^–7^	2.70 × 10^–7^	2.70 × 10^–7^	2.70 × 10^–7^

This case study also confirm sthat the proposed formulation can accurately recover the ground-truth parameters. All solvers converge to identical objective values (2.70 × 10^–7^), indicating that this problem is well-conditioned. Standard solvers like CONOPT, IPOPT, and GUROBI achieved this solution in approximately 0.12 s. Our work remains highly computationally competitive with 0.379 s which is twice as fast as the global solver BARON (0.628 s).

### 4.3 Ethane Dehydrogenation (EDH) Gas-Phase Network in a CSTR

To extend the proposed method to gas-phase reaction networks, the third case study considers an Ethane Dehydrogenation (EDH) system.^39^ The network involves seven components and three primary reactions: Ethane Dehydrogenation (EDH), Ethane Hydrogenolysis (HYD), and the Reverse Water–Gas Shift (RWGS) reaction. This study adapts the system to a CSTR environment to generate synthesized concentration profiles. Compared with the previous examples, this system contains a larger number of kinetic parameters (9) and introduces stronger nonlinear coupling between reactions. As a result, the estimation problem becomes more challenging. The reaction network is defined by the following overall reactions:

$$\mathrm{EDH}:C_{2}H_{6}⇌C_{2}H_{4}+H_{2}$$

$$\mathrm{HYD}:C_{2}H_{6}+H_{2}⇌2{\mathrm{CH}}_{4}$$

$$\mathrm{RWGS}:{\mathrm{CO}}_{2}+H_{2}⇌\mathrm{CO}+H_{2}O$$

The net reaction rate for reaction *i* is modeled using a thermodynamic driving-force form:

30 $$r_{\mathrm{net},i}=r_{\mathrm{fwd},i}\left(1-\frac{Q_{i}}{K_{c,i}}\right)$$

where *Q*_*i*_ is the reaction quotient and *K*_*c,i*_ is the equilibrium constant. The forward rate follows an Arrhenius relationship:

31 $$r_{\mathrm{fwd},i}=A_{0,i\;}\exp\left(-\frac{E_{a,i}}{RT}\right)\prod \left[\mathrm{reactants}\right]$$

where the bracketed term denotes the product of reactant concentrations for reaction *i*. For the EDH gas-phase reaction network, the dynamic CSTR balances are constructed from the stoichiometry of the EDH, HYD, and RWGS reactions together with the rate expressions in eqs 40–41. In the parameter estimation problem, these balances are imposed at steady state by setting the time derivatives of all gas-phase species concentrations equal to zero. The resulting algebraic equations define the constraint vector *h*(*x*,*p*,*r*) = 0 in eq 22 for this case study. Synthetic data were generated by simulating at four temperatures: 835 K, 848 K, 861 K, and 873 K. The inlet feed composition was set to C_2_H_6_ = 1.0 mol/L, H_2_ = 0.5 mol/L, and CO_2_ = 0.5 mol/L, with all other inlet species set to zero. The parameter estimation task aims to recover 9 unknown parameters: the pre-exponential factors $\{A_{0,i}{\}}_{i=1}^{3}$ , activation energies $\{Ea_{i}{\}}_{i=1}^{3}$ , and equilibrium constants $\{K_{c,i}{\}}_{i=1}^{3}$ for all three reactions. To assess robustness, two different sets of parameter bounds were used: loose bounds and tight bounds. For activation energies, loose and tight bounds were same. The model parameters used as ground-truth and bounds on the parameters are summarized in Table 5.

**Table 5:** Parameter Bounds Used in the Optimization Experiments^a^

Parameter	Reaction	Ground Truth (GT)	Loose Bounds	Tight Bounds
*A*	*r*_1_	1.0 × 10^8^	[5.0 × 10^6^, 2.0 × 10^9^]	[8.0 × 10^7^, 1.2 × 10^8^]
	*r*_2_	5.0 × 10^7^	[2.5 × 10^6^, 1.0 × 10^9^]	[4.0 × 10^7^, 6.0 × 10^7^]
	*r*_3_	1.0 × 10^7^	[5.0 × 10^5^, 2.0 × 10^8^]	[8.0 × 10^6^, 1.2 × 10^7^]
*Ea*	*r*_1_	120000	[100000, 140000]	[100000, 140000]
	*r*_2_	100000	[80000, 120000]	[80000, 120000]
	*r*_3_	80000	[60000, 100000]	[60000, 100000]
*K*_*c*_	*r*_1_	0.5	[0.05, 5.0]	[0.40, 0.60]
	*r*_2_	1.2	[0.12, 12.0]	[0.96, 1.44]
	*r*_3_	0.8	[0.08, 8.0]	[0.64, 0.96]

a Loose bounds allow a wide search space around the Ground Truth (GT), while tight bounds restrict the parameter range to values close to the GT.

To further investigate the sensitivity of the estimation problem to initialization and parameter bounds, we repeated the optimization using 1000 randomly generated initial guesses within the specified parameter bounds. All solvers (our algorithm, IPOPT, CONOPT, BARON, and GUROBI) were initialized with identical starting points to ensure a fair comparison. For each run, the deviation of the estimated parameters from the ground truth values was recorded. Note that GUROBI did not return feasible solutions within a 3600 s time limit.

Figures 1 and 2 summarize the deviation scenarios of the estimated parameters from the ground-truth values across all runs. Several important observations emerged from these numerical experiments. First, when wide bounds are used, the estimation problem exhibits strong solution multiplicity. Multiple parameter combinations produce similar objective values and concentration profiles, resulting in a widespread of estimated parameters across the runs. This effect is particularly pronounced for the pre-exponential factors *A*_i_, whose large magnitudes make the estimation problem highly sensitive to scaling and nonlinear coupling between reactions. As a consequence, the deviations for *A*_i_ are significantly larger than those observed for activation energies and equilibrium constants. Second, tightening the parameter bounds substantially reduces the variability of the estimated parameters. The spread of the solutions decreased markedly for all solvers. This result highlights the importance of incorporating prior physical knowledge on the system, that is, estimates obtained from density functional theory (DFT), thermodynamic constraints, or literature data when solving large nonlinear parameter estimation problems. Without such information, the optimizer may converge to multiple parameter sets that reproduce the observed data equally well but lack a physical interpretability. Third, although the bounds on activation energies were kept identical in both the loose- and tight-bound scenarios, their estimated variability is significantly reduced under tighter bounds on the pre-exponential factors and equilibrium constants. This behavior reflects the strong coupling among kinetic parameters.

**Figure 1:**
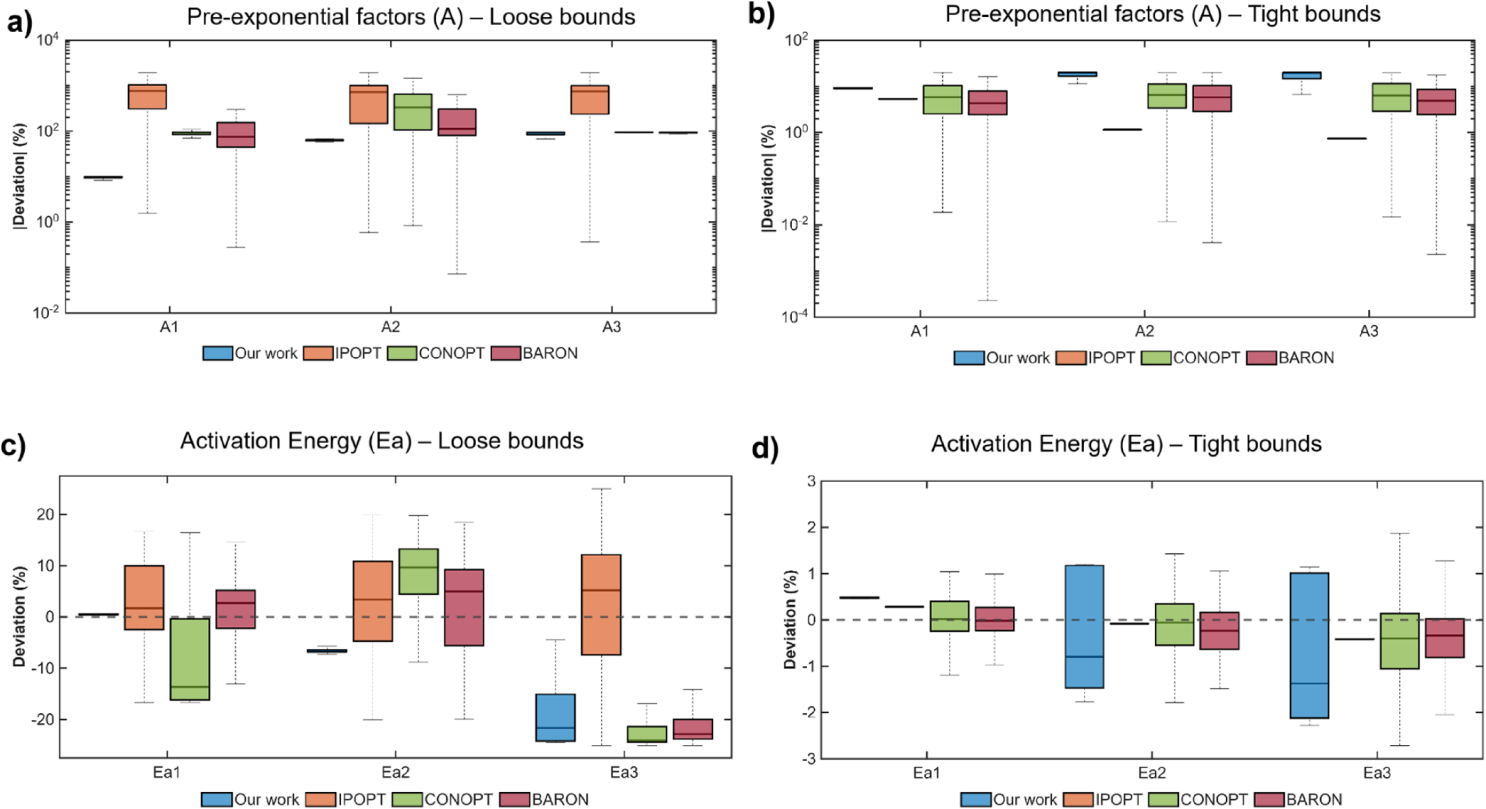
Distribution of estimated kinetic parameters across 1000 runs under loose and tight parameter bounds. Tightening the bounds significantly reduces the spread of the estimated parameters.

**Figure 2:**
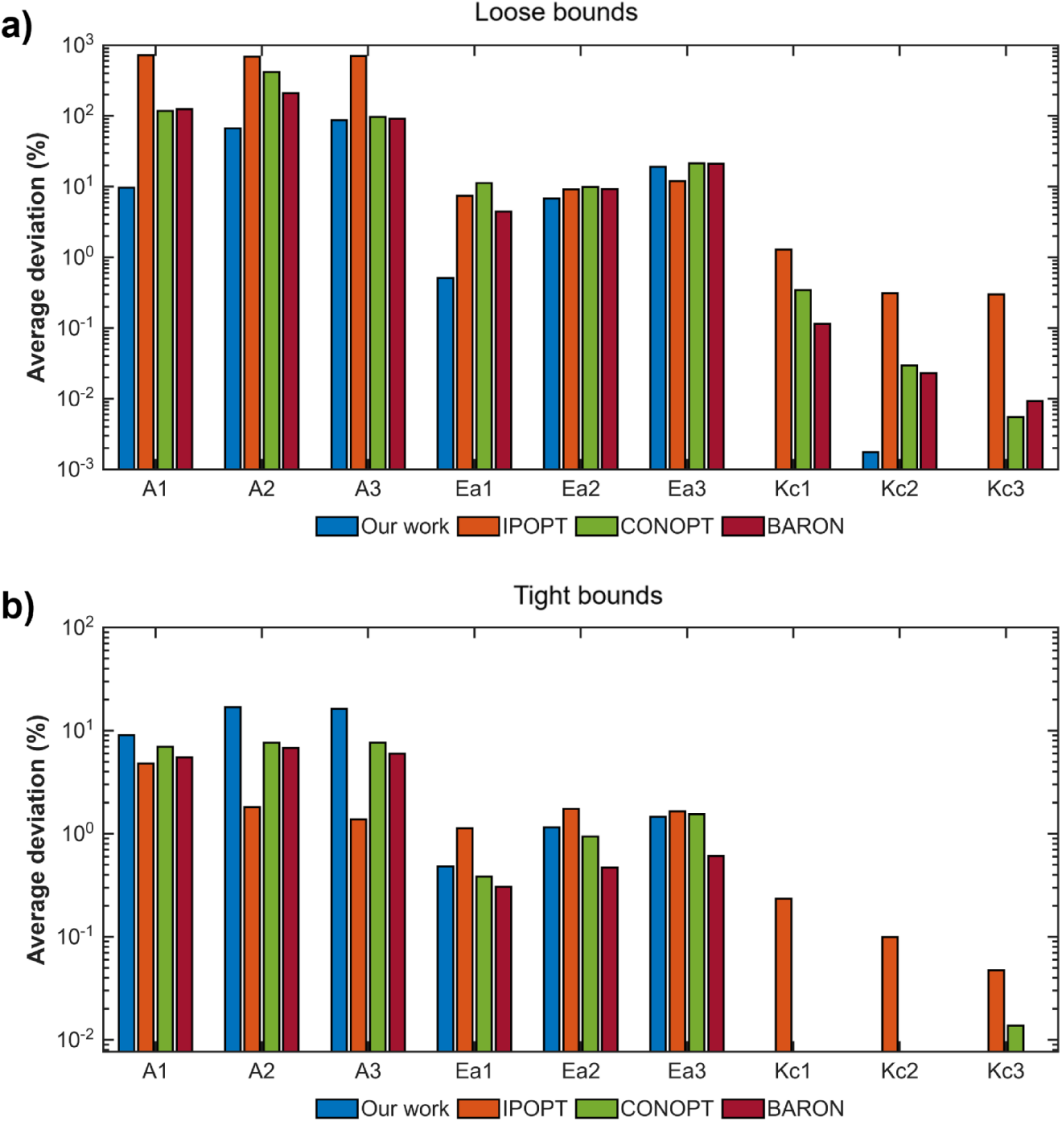
Comparison of average deviation from ground truth for different solvers across all parameters: (a) Average parameter deviation across all iterations with loose bounds, (b) Average parameter deviation across all iterations with tight bounds. Tight parameter bounds substantially reduce estimation error and solver variability.

Fourth, the solver comparison reveals clear differences in robustness. The proposed algorithm consistently identifies solutions close to the ground-truth parameters and maintains relatively stable estimates across multiple runs, even with loose bounds. Although the deviations for the pre-exponential factors are somewhat larger for this approach compared with some global solvers, they remain within approximately ± 20% of the true values. BARON demonstrates strong robustness in several cases but exhibits larger spreads in certain parameters across repeated runs. In contrast, IPOPT frequently converges to poorer local solutions and displays the largest deviations across most parameters. CONOPT performs moderately well but shows noticeable variability when the parameter bounds are wide. Finally, the equilibrium constants *K*_*c,i*_ exhibit significantly smaller deviations compared with the kinetic parameters. Since their magnitudes are closer to unity and their influence on the rate expressions is less strongly amplified, these parameters are easier to recover accurately, even under wide parameter bounds. Overall, these results highlight the inherent nonconvexity and parameter correlation present in nonlinear reaction network estimation problems. The numerical experiments demonstrate that bound tightening plays a critical role in reducing solution multiplicity. For realistic microkinetic models involving large numbers of parameters, incorporating physically informed parameter bounds is therefore essential to obtain meaningful and interpretable kinetic estimates.

The summary of the solver performance is shown in the following Table 6 based on 1000 initial guesses. The results demonstrate that the proposed algorithm achieves the highest robustness and accuracy across the 1,000 initial guesses, yielding the lowest average sum of squared errors (SSE) of 3.278 × 10^–10^. While standard local solvers like IPOPT and CONOPT exhibit faster execution times (under 210 s), their higher average SSE values indicate a tendency to converge to inferior local minima. Conversely, our method delivers global-level accuracy and slightly outperforms the global solver BARON while reducing the computational time by approximately 32%. Note that GUROBI did not return feasible solutions within the 3600 s time limit.

**Table 6:** Solver Performance Comparison for EDH Case Study

Metric	Our Work	IPOPT	CONOPT	BARON
Time to solve 1000 iterations (s)	418	206.297	157.776	617.009
Average SSE	3.278 × 10^–10^	9.58 × 10^–7^	2.66 × 10^–9^	3.578 × 10^–10^

It should be noted that a low objective value does not necessarily imply unique recovery of the underlying kinetic parameters. Because kinetic parameter estimation is nonconvex and often practically nonidentifiable, multiple local parameter sets may reproduce similar concentration, conversion, or selectivity data while implying different mechanistic interpretations. In the synthetic EDH case study, the availability of ground-truth parameters allows this issue to be examined directly by comparing the estimated parameters against the true values over multiple initializations. The results show that loose bounds allow a larger parameter spread, whereas tighter, physically informed bounds substantially reduce deviation from the ground-truth values and solver variability. Thus, bound tightening should not be interpreted as proving uniqueness; rather, it restricts the solution space to a physically plausible neighborhood where the inferred kinetic behavior is less likely to change qualitatively.^76^ For experimental systems, where true parameters are unavailable, the estimated parameters should be interpreted as physically constrained calibrated parameter sets. Future work will further assess whether different local solutions imply different chemistries by comparing reaction fluxes, surface coverages, rate-controlling steps, reaction orders, and apparent activation energies across multiple fitted parameter sets.

### 4.4 Applications to Microkinetic Models

For the MKM case studies in this section, the constraint vector *h*(*x*,*p*,*r*) = 0 is defined using the MKM formulation in eqs 9–16. This includes the gas-phase CSTR balances, surface-species balances, site balance, elementary step rate expressions, and temperature-dependent rate constant definitions.

#### 4.4.1 Catalytic CO Oxidation

Before applying the proposed parameter estimation method to complex catalytic systems, a controlled benchmark problem was constructed using a synthetic microkinetic model. The purpose of this example is to evaluate the ability of different optimization solvers to recover known kinetic parameters when accurate ground truth values and physically consistent bounds are available. In this case study, the kinetic parameters are assumed to be known a priori (e.g., from density functional theory calculations), and synthetic reactor data are generated using these parameters. The parameter estimation problem then attempts to recover these known parameters from generated data.

The system corresponds to the catalytic oxidation of carbon monoxide:

32 $$2CO+O_{2}\to 2CO_{2}$$

This reaction is of significant industrial relevance, particularly in automotive catalytic converters where CO must be efficiently oxidized to reduce harmful emissions. The overall reaction is represented using a microkinetic description consisting of eight elementary surface reactions.^2^ Vacant catalytic sites are denoted by *, while adsorbed species are represented with the superscript *.

CO+*→CO*

CO*→CO+*

O2+2*→2O*

2O*→O2+2*

CO*+O*→CO2*+*

CO2*+*→CO*+O*

CO2+*→CO2*

CO2*→CO2+*

The kinetic rate constant of each elementary step *j* is described using the modified Arrhenius expression:

$$k_{j}=A_{j}T^{b_{j}}\exp\left(-\frac{{Ea}_{j}}{RT}\right)$$ 33

where *A*_*j*_ denotes the pre-exponential factor, *b*_*j*_ is the temperature exponent, and *Ea*_*j*_ is the activation energy. The universal gas constant and temperature are represented by *R* and *T*, respectively. For catalytic CO oxidation, eqs 13–14 are replaced by eq 43. To generate synthetic experimental data, the microkinetic model was implemented as a system of ordinary differential equations describing species balances in a CSTR. The reactor configuration was characterized by a surface-to-volume ratio of *A* ^*sur*^/*V* = 10^4^ and a residence time τ = 1. The system was simulated using MATLAB until steady-state conditions were achieved. The resulting steady-state gas-phase concentrations were treated as synthetic experimental observations. The parameter estimation problem therefore attempts to recover the original kinetic parameters used in the data generation step. It simultaneously determines three kinetic parameters for each elementary reaction: *A*_*j*_, *b*_*j*_ and *Ea*_*j*_. Since the mechanism contains eight elementary reactions, the estimation problem involves a total of 24 kinetic parameters. The objective function minimizes the sum of squared errors (SSE) between predicted and synthetic experimental concentrations. For BARON, additional numerical adjustments were required in order to obtain feasible solutions. Specifically, the activation energy parameters had to be scaled by 10^5^ to avoid numerical infeasibility. Furthermore, the feasibility tolerance had to be relaxed to 5 × 10^–5^ in order to allow convergence. Without these modifications, BARON consistently reported the problem as infeasible. These adjustments highlight the numerical challenges associated with solving highly nonlinear microkinetic parameter estimation problems using global optimization solvers.

Figure 3 provides a comprehensive evaluation of solver performance, revealing the critical disconnect between mere mathematical convergence and true parameter recovery. Note that GUROBI is excluded from these visualizations as it failed to identify a feasible solution within the 3,600 s time limit, despite extensive manual tolerance relaxation and parameter scaling. As illustrated in the box plot (Figure 3a), the proposed solver consistently enforces the tightest error bounds on parameter deviations. Our formulation restricts the median deviation to strictly below 10% with a compressed interquartile range indicating uniform reliability. In contrast, local solvers such as CONOPT and IPOPT exhibit median deviations of nearly 15% and 30%, respectively, accompanied by erratic upper quartiles. The global solver BARON suffers from catastrophic parameter drift, yielding a median deviation reaching 100%.

**Figure 3:**
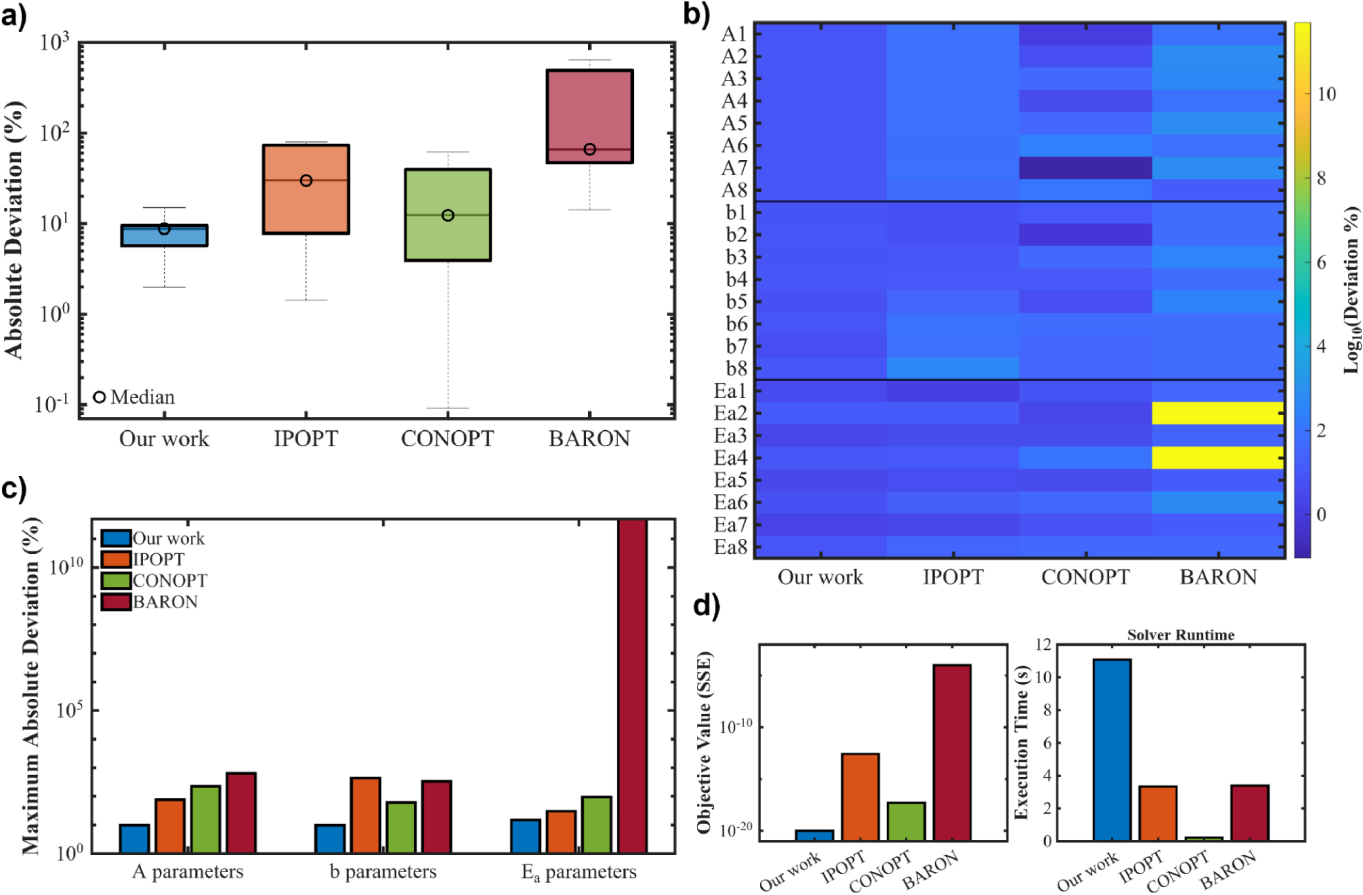
Comprehensive comparison of solver performance and parameter deviations for the microkinetic model. (a) Boxplot showing the overall distribution of deviations; (b) Heatmap detailing individual parameter errors; (c) Maximum absolute deviation categorized by parameter class; and (d) Overall objective value and computational runtime.

A parameter-wise heatmap of the estimation deviations is presented in Figure 3B. The heatmap highlights the sensitivity of the optimization problem to the activation energy parameters. In particular, BARON produces extremely large deviations for *Ea*_2_ and *Ea*_4_, which exceed several orders of magnitude relative to the ground-truth values. To further analyze solver performance, the deviations were grouped according to parameter type, as shown in Figure 3c. Our algorithm successfully bounds the maximum deviation across pre-exponential factors (*A*), temperature exponents (*b*), and activation energies (*E*_a_) to near 10%, while competing solvers exhibit severe vulnerabilities.

Finally, Figure 3d highlights a common pitfall in nonlinear kinetic fitting: the tendency of solvers to converge to low-residual local minima that lack physical accuracy. While IPOPT and CONOPT rapidly minimize the sum of squared errors (reaching approximately 10^–13^ and 10^–17^ in under 4 s), panels (a)–(c) demonstrate that they still yield highly deviated parameter sets. In contrast, the proposed solver reduces the objective value to 10^–20^. Although the system is inherently nonunique and does not allow exact parameter recovery, the proposed KKT residual-correction algorithm effectively limits unphysical parameter drift. As a result, the estimated parameters remain close to the ground-truth values. The execution time of approximately 11 s represents a practical trade-off for achieving stable and physically meaningful solutions in this structurally underdetermined problem.

This case study shows that even relatively small reaction networks can produce highly nonlinear optimization landscapes. As a result, standard nonlinear optimization solvers can converge to parameter sets that reproduce the data but deviate significantly from the true kinetic parameters. Moreover, global optimization solvers, such as BARON and GUROBI, may experience numerical difficulties when large parameter ranges are present. In this case study, activation energy scaling and tolerance relaxation were required to obtain feasible solutions. Such adjustments are rarely feasible in large microkinetic models where the parameter magnitudes vary across multiple orders. Therefore, the proposed solver demonstrates strong robustness and parameter recovery accuracy without requiring solver-specific modifications.This suggests that our work is well-suited for high-dimensional microkinetic parameter estimation problems. The next section extends this analysis to three catalytic systems.

#### 4.4.2 Parameter Estimation Based on Experimental Data: NO Reduction, Ammonia Decomposition, and CO_2_ Hydrogenation

To evaluate the robustness and generality of the proposed parameter estimation algorithm, three reaction networks of increasing complexity were considered: reduction of NO by CO on Pt catalyst (System 1), ammonia decomposition on Ni catalyst (System 2), and CO_2_ hydrogenation to methanol on In_2_O_3_ (System 3). These case studies differ in reaction topology, number of parameters, number of species, and the amount of experimental data available for parameter estimation. The summary of these systems is provided in Table 7:

**Table 7:** Summary of the Reaction Systems Used in This Study

Metric	System 1 (NO reduction)	System 2 (Ammonia decomposition)	System 3 (CO_2_hydrogenation)
Total Steps	9 ^50^	12 ^77,78^	26 ^79^
Gas Species	5	3	5
Data Points	15 ^50,80^	9 ^77,81^	3 ^82^
Parameters to Estimate	23	30	65

The reaction mechanisms are provided in the Supporting Information. For the initial guesses, pre-exponential factors (*A*) were set to a value of 1 × 10^13^ s^–1^ which is generally acceptable according to transition state theory for surface reactions.^1,20,83^ For activation energies, we have depended on literature-based data (i.e, from DFT, experiments, semiempirical relationships, etc.) for initial guesses and parameter bounds. As sticking coefficients should be 0 to 1, the initial guesses were set to 0.1 for them. Across all systems, kinetic parameters were estimated using our algorithm and compared against widely used nonlinear optimization solvers, including BARON, IPOPT, and CONOPT. Gurobi was unable to find any feasible solutions after 3600 s runtime for any of these systems. We also compare our results with a traditional ODE-based optimization approach using FMINSEARCH, where parameter estimation is carried out through repeated ODE integration rather than direct NLP solution.

Table 8 highlights clear performance disparities among the solvers as problem complexity scales. In System 1, IPOPT and CONOPT converge rapidly (1–1.4 s) but yield unacceptably high errors (SSE of 10^7^–10^8^), indicating poor parameter recovery despite apparent convergence. BARON fails to find a feasible solution. The proposed method, however, reduces the error to 1 × 10^–9^ within a moderate 31.6 s, significantly outperforming the alternatives. The FMINSEARCH solver, representing the traditional MKM parameter estimation workflow of repeatedly integrating the underlying ODEs rather than solving a direct NLP, occasionally provides more reliable parameter estimates than conventional NLP solvers. But integrating stiff ODEs at each iteration incurs a severe computational penalty, driving runtimes to 169 s for System 1 and over 1200 s for System 2. For System 2, all solvers locate feasible points, but the SSE widens drastically. IPOPT and BARON stall at an SSE of 27–28, whereas the proposed method shrinks the error by over 8 orders of magnitude (to 10^–7^) while maintaining a highly competitive runtime. This contrast peaks in the high-dimensional landscape of System 3. Here, IPOPT and CONOPT become trapped in high-error region (SSE of 10^4^–10^5^), and BARON fails after 884 s of computational effort. The proposed algorithm effortlessly maintains feasibility and high accuracy (SSE of 10^–13^) in just 7.7 s. These results demonstrate that as nonlinearity and dimensionality increase, conventional solvers either fail to locate feasible regions or converge to physically inaccurate parameter sets. The proposed approach, however, delivers consistently robust and accurate performance across all tested complexities.

**Table 8:** Summary of Solver Performance Across the Three Reaction Systems

System	Solver	SSE	Execution Time (s)
System 1	BARON	Infeasible	20.296
	CONOPT	1.76 × 10^8^	1.116
	IPOPT	1.078 × 10^7^	1.399
	Our work	1 × 10^–9^	31.65
	FMINSEARCH	1.50 × 10^3^	169.23
System 2	BARON	27.883	3.861
	CONOPT	11.011	1.006
	IPOPT	27.870	1.865
	Our work	1.02 × 10^–7^	28.983
	FMINSEARCH	3.49	1220.07
System 3	BARON	Infeasible	884
	CONOPT	1.95 × 10^5^ ^a^	0.247
	IPOPT	1.20 × 10^4^ ^a^	3.784
	Our work	2.91 × 10^–13^	7.70
	FMINSEARCH	6.92 × 10^4^	113.20

a This indicates locally infeasible solutions.

While it is desirable to reduce SSE, a typical parameter estimation may not reduce the SSE to zero in the presence of noise, large data sets, and model errors. However, when analyzing our results, we see that the SSE values are very small, which indicates potential overfitting. This overfitting can be a result of an underdetermined system when the number of adjustable kinetic parameters exceeds the number of independent observations. A possible remedy is to include additional experimental data. Another point to consider is that the ground-truth kinetic parameters are available only for the synthetic case studies. For experimental systems, the estimated parameters should be interpreted as physically constrained calibrated parameter sets rather than as unique ground-truth values.

Figure 4 compares predicted gas concentrations with experimental observations for system 1. The proposed algorithm accurately reproduces all species profiles over the entire temperature range, capturing both the rapid depletion of NO and CO and the formation of CO_2_, N_2_, and N_2_O. While FMINSEARCH and IPOPT also successfully capture these overall nonlinear dynamics with some deviations, CONOPT fails entirely to locate the reaction pathways. These discrepancies arise from the strong coupling between surface intermediates and gas-phase species, which create a highly nonlinear optimization landscape with multiple local minima.

**Figure 4:**
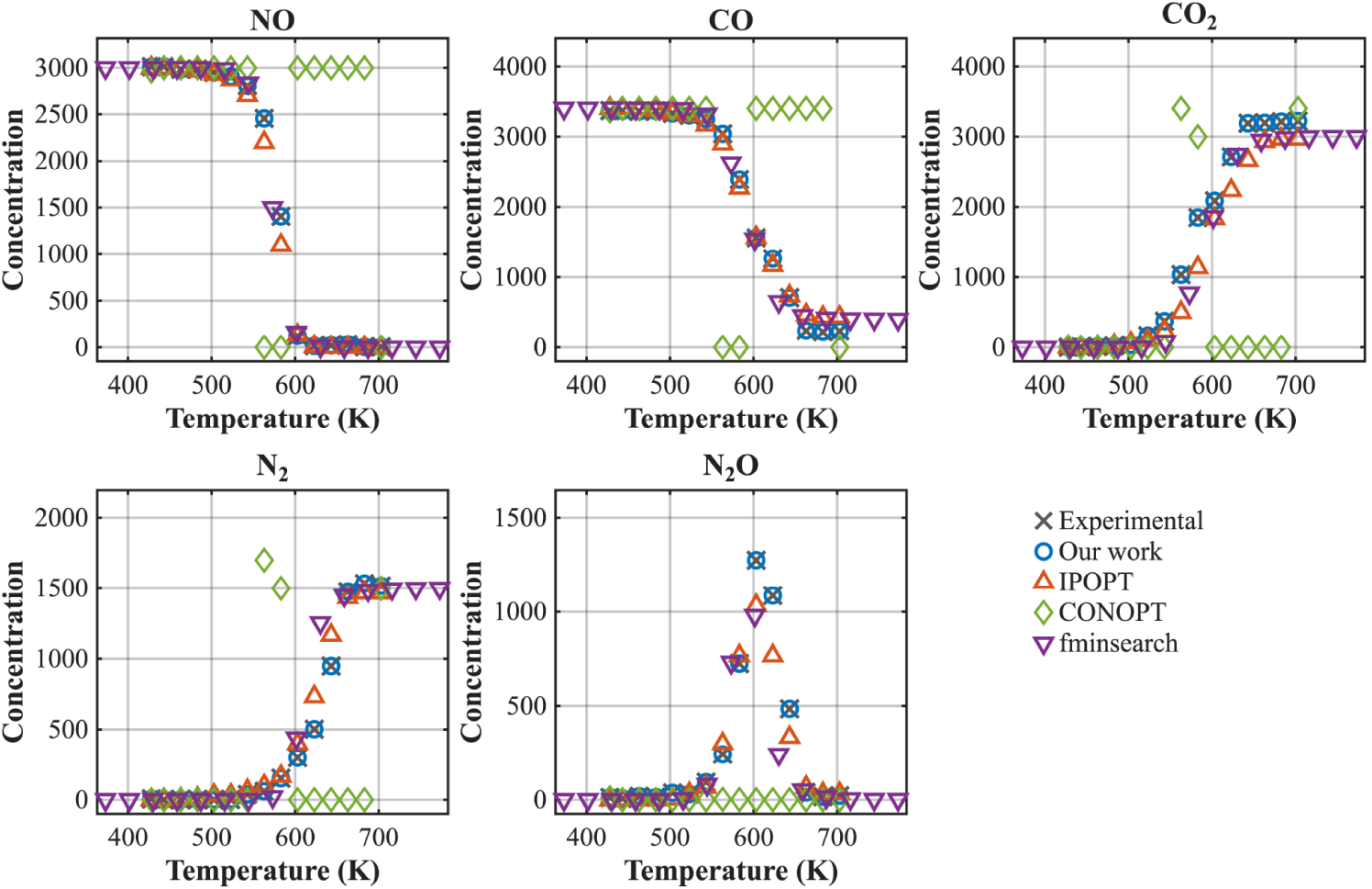
Predicted and experimental species concentration profiles for the NO reduction by CO system.

As shown in Figure 5, the proposed algorithm accurately reproduces the experimental ammonia conversion curve across the entire temperature range. Conventional solvers, BARON and IPOPT, fail to capture the nonlinear temperature dependence of the conversion, instead converging to trivial near-zero solutions. CONOPT yields highly inconsistent results that fail to capture the trend, while FMINSEARCH consistently underestimates the high-temperature conversion plateau. This behavior reflects the strong stiffness present in the underlying microkinetic ODE system. Small variations in kinetic parameters can dramatically alter surface coverages and reaction rates, making the estimation problem extremely sensitive to initialization and numerical conditioning.

**Figure 5:**
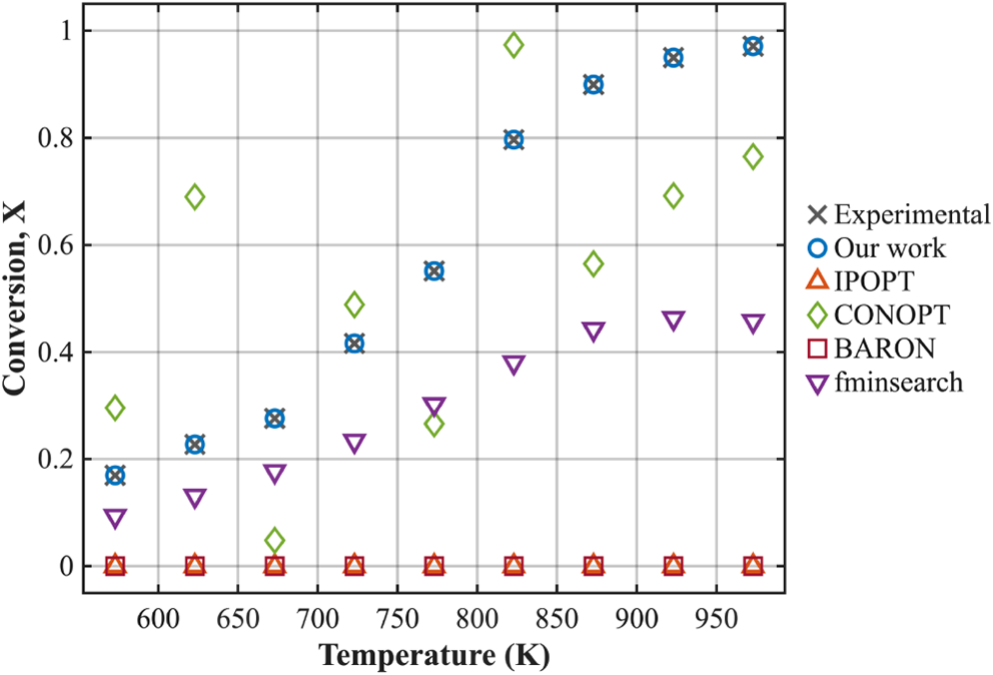
Ammonia conversion as a function of temperature. The proposed method successfully reproduces the nonlinear conversion behavior observed experimentally.

Figure 6 presents the predicted and experimental species concentrations for the methanol synthesis system. Our work successfully reconstructs the observed concentration profiles across all measured species, demonstrating its capability of handling larger reaction networks with strongly coupled kinetics. Both IPOPT and CONOPT produce scattered, inaccurate concentration profiles, struggling notably to reproduce the formation pathways of CO_2_, H_2_O, and CO. Meanwhile, the FMINSEARCH algorithm becomes trapped in a suboptimal local minimum.

**Figure 6:**
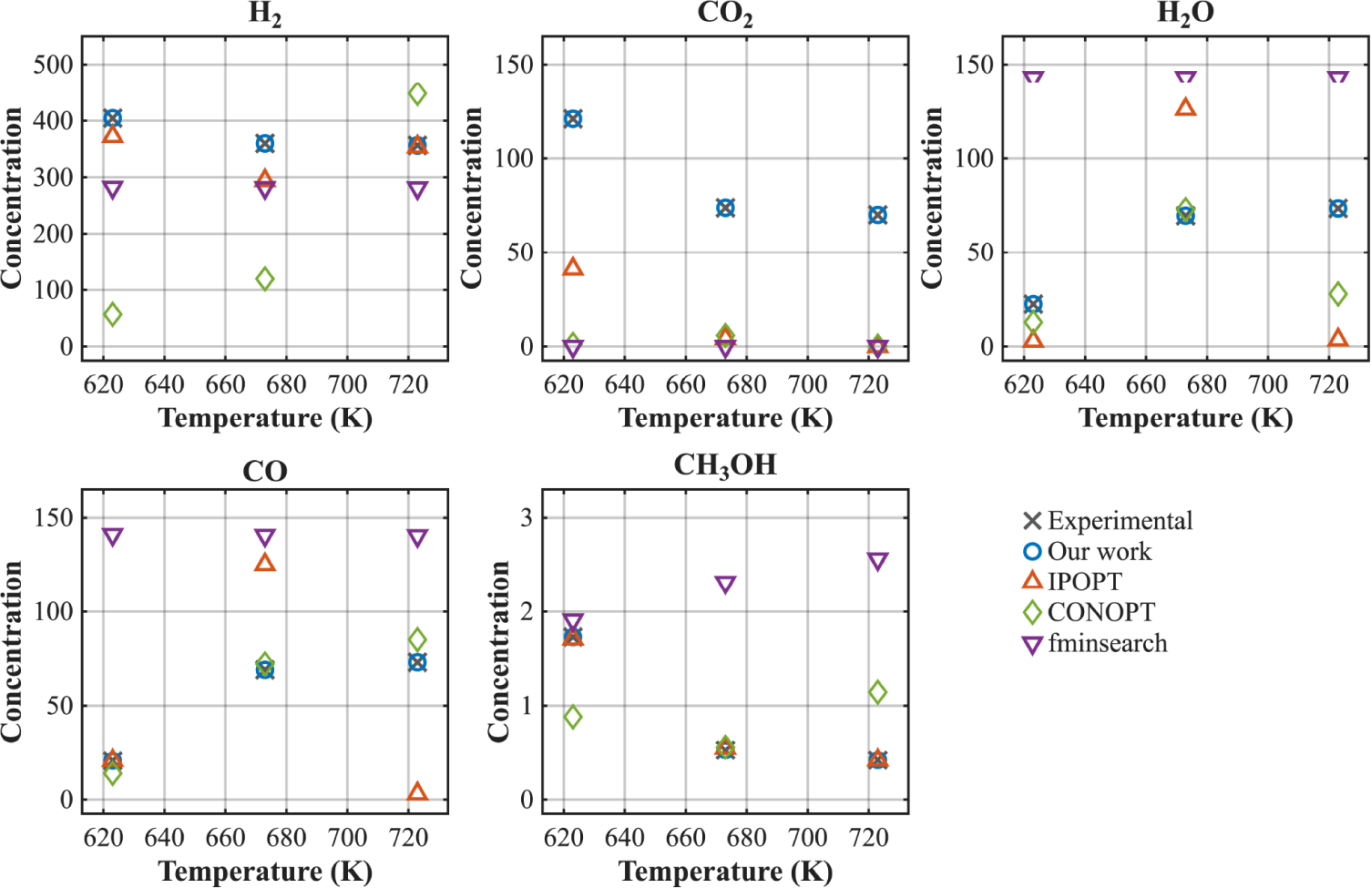
Predicted and experimental species concentration profiles for the CO_2_ hydrogenation to methanol system.

Figure 7 provides a direct comparison of predictive accuracy across solvers. The proposed method achieves perfect agreement with the parity line in all three systems (*R* ^2^ = 1.000), confirming exact reconstruction of the experimental data. In contrast, conventional solvers exhibit substantial scatter and loss of predictive consistency. For System 1, CONOPT shows severe deviation with negative correlation (*R* ^2^ = −0.284), while IPOPT and FMINSEARCH achieve good agreement (*R* ^2^ ≈ 1). For System 2, IPOPT and BARON yield bad fits with negative *R* ^2^ values (−3.604), indicating failure to capture the underlying trend. In System 3, *R* ^2^ drops to 0.859 (IPOPT), 0.209 (CONOPT), and 0.627 (FMINSEARCH). These results demonstrate that while conventional solvers may converge numerically, they fail to produce reliable predictions, whereas the proposed approach consistently preserves both numerical accuracy and physical fidelity across all systems.

**Figure 7:**
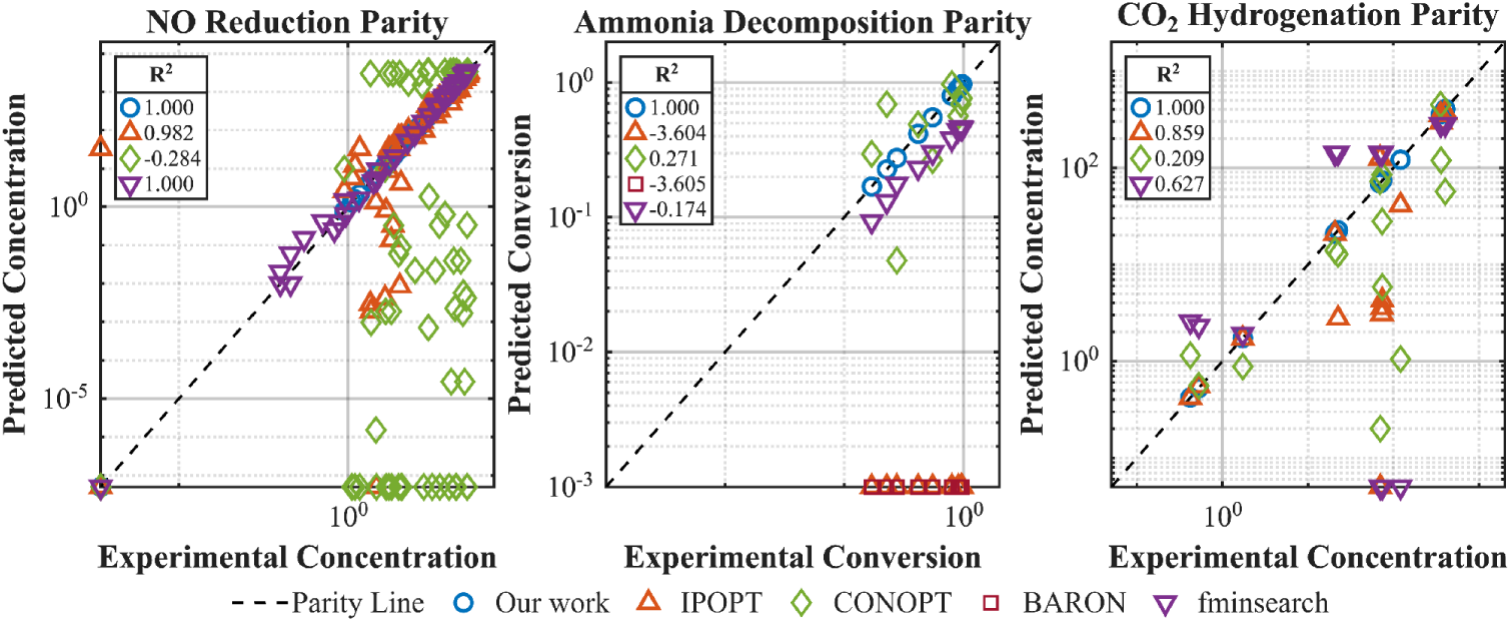
Parity plots comparing predicted and experimental observations across the three case studies.

The three case studies demonstrate the robustness and scalability of the proposed parameter-estimation methodology. Despite substantial differences in reaction network size, data availability, and parameter dimensionality, the method consistently identifies parameter sets capable of reproducing experimental observations with a very small residual error. The results also highlight a fundamental limitation of traditional optimization-based approaches to microkinetic parameter estimation. The highly nonlinear coupling among kinetic parameters, surface coverages, and gas-phase species creates a challenging optimization landscape that conventional solvers struggle to navigate. As the dimensionality of the parameter space increases, these difficulties become more pronounced, often leading to infeasible solutions or convergence to poor local minima. The proposed approach effectively overcomes these challenges. Consequently, it provides a reliable method for parameter estimation, even in large and strongly nonlinear catalytic reaction networks.

The results in this section show that the proposed framework can estimate parameter sets that reproduce the available experimental observations under imposed physical bounds. However, these results should be interpreted with two different sources of nonuniqueness in mind: parameter degeneracy and steady-state solution multiplicity.^50,84–86^ Parameter degeneracy means that multiple kinetic parameter sets may reproduce similar experimental observables. In contrast, steady-state multiplicity means that for a fixed parameter set, the nonlinear CSTR algebraic equations may admit multiple concentration or surface coverage solutions. Such multiplicity can occur in catalytic systems with strong adsorption, surface poisoning, ignition, and extinction behavior, or other nonlinear feedback mechanisms. Therefore, an algebraic steady-state solution does not by itself prove uniqueness, dynamic reachability, or stability. In this work, the estimated parameter sets were checked by integrating the corresponding dynamic CSTR equations from the physically meaningful initial conditions. For the systems considered, the transient simulations converged to steady-state concentrations consistent with the algebraic NLP solutions. Future work will incorporate local stability analysis and bifurcation or continuation methods to track multiple steady-state branches and identify dynamically stable physically relevant solutions.

## 5 Conclusion

In this work, we developed a generalized algorithm for kinetic parameter estimation in MKMs by solving the steady-state algebraic system using a KKT-based method. The proposed formulation improves numerical conditioning and enables the integration of both molecular-scale information obtained from DFT and process-scale experimental observations. Through several case studies, including the reduction of NO by CO on pt, NH_3_ decomposition on Ni, and CO_2_ hydrogenation to methanol over In_2_O_3_, our approach successfully identified kinetic parameters capable of reproducing the observed experimental behavior. The results also highlight the importance of physically informed parameter bounds. Incorporating physically informed parameter bounds (i.e, from experimental measurements, DFT calculations, Brønsted–Evans–Polanyi (BEP) relations, and other semiempirical scaling relationships) significantly enhances the robustness and convergence of the estimation procedure by constraining the search space to physically meaningful regions. In contrast, conventional nonlinear programming solvers frequently struggle with the highly nonlinear and ill-conditioned optimization landscape characteristic of MKM parameter estimation problems, often converging to infeasible or poor local solutions. The proposed approach consistently achieves stable convergence in cases where traditional solvers fail to recover physically meaningful parameter sets. The current formulation does not explicitly enforce thermodynamic constraints; however, incorporating such constraints in future work may further improve thermodynamic consistency, albeit at the cost of increased computational complexity. Multiplicity of the solutions further reveals that the globally optimal solution is not always the most physically meaningful choice in catalyst modeling. Instead, identifying parameter sets that remain consistent with physical bounds and experimental observations is more relevant for practical catalytic analysis. Overall, the proposed algorithm provides a systematic and robust approach for estimating MKM parameters.

## Supplementary Material



## Notes

This material is available free of charge via the Internet at Illustrative example of the model formulation, definitions of the KKT algorithmic parameters, and elementary reaction mechanisms for three systems (PDF)
