# Supplementary material for: Parameter Estimation of Microkinetic Models for Heterogeneous Catalysis
Source: Ind Eng Chem Res. 2026 Jul 15;65(30):16139–56. doi: 10.1021/acs.iecr.6c01417 (PMC13455557; doi:10.1021/acs.iecr.6c01417)
Supplement: Supplementary file 1 [file ie6c01417_si_001.pdf]

Parameter Estimation of Microkinetic Models for Heterogeneous Catalysis

M G Toufik Ahmed[1], **Bimol Nath Roy**[1], **and M. M. Faruque Hasan**[1,2,*]
[1]Artie McFerrin Department of Chemical Engineering, Texas A&M University, College Station, TX 77843-3122, USA.
[2]Texas A&M Energy Institute, Texas A&M University, College Station, TX, 77843, USA.

Contents

S1 Illustrative Example **S2**

S2 Reaction Mechanism **S5**

List of Tables

S1 Definitions of algorithmic parameters used in the KKT-based iterative method. . . . S5
S2 Elementary reaction mechanisms for the three benchmark systems S5

S1 Illustrative Example

To demonstrate the model structure, consider a simplified hypothetical reaction network involving three gas species (A, B_2, C), one vacant site (*), and three surface-bound intermediates (A*, B*, C*). The overall reaction is

$$A + B_2 \rightarrow C$$

In the present formulation, each forward and reverse event is treated as a separate irreversible elementary step. The resulting reaction network consists of eight irreversible reactions:

$$\begin{aligned}
&(1) \quad A + * \rightarrow A* \\
&(2) \quad A* \rightarrow A + * \\
&(3) \quad B_2 + 2* \rightarrow 2B* \\
&(4) \quad 2B* \rightarrow B_2 + 2* \\
&(5) \quad A * + B* \rightarrow C * + * \\
&(6) \quad C * + * \rightarrow A * + B* \\
&(7) \quad C + * \rightarrow C* \\
&(8) \quad C* \rightarrow C + *
\end{aligned}$$

The species are indexed as

$$\mathcal{I} = \{1, 2, 3, 4, 5, 6, 7\}$$

with the corresponding ordering

$$[1, 2, 3, 4, 5, 6, 7] = [A, B_2, C, *, A*, B*, C*]$$

The gas, vacant-site, and adsorbed-intermediate subsets are

$$\mathcal{I}_G = \{1, 2, 3\}, \qquad \mathcal{I}_S = \{4\}, \qquad \mathcal{I}_I = \{5, 6, 7\}$$

The reaction index set is

$$\mathcal{R} = \{1, 2, 3, 4, 5, 6, 7, 8\}$$

For this example, the adsorption-step subset and surface-reaction subset are defined as

$$\mathcal{R}_{ads} = \{1, 3, 7\}, \qquad \mathcal{R}_{sur} = \{2, 4, 5, 6, 8\}$$

The participation indicator matrix $\varepsilon_{i,j}$ identifies the role of species i in reaction j, where entries

are strictly defined as -1 for a reactant, 1 for a product, and 0 if the species does not participate:

$$\varepsilon_{i,j} = \begin{bmatrix} -1 & 1 & 0 & 0 & 0 & 0 & 0 & 0 \\ 0 & 0 & -1 & 1 & 0 & 0 & 0 & 0 \\ 0 & 0 & 0 & 0 & 0 & 0 & -1 & 1 \\ -1 & 1 & -1 & 1 & 1 & -1 & -1 & 1 \\ 1 & -1 & 0 & 0 & -1 & 1 & 0 & 0 \\ 0 & 0 & 1 & -1 & -1 & 1 & 0 & 0 \\ 0 & 0 & 0 & 0 & 1 & -1 & 1 & -1 \end{bmatrix}$$

The absolute stoichiometric matrix $\nu_{i,j}$ defines the total number of molecules of species i participating in reaction j, regardless of whether they are reactants or products:

$$\nu_{i,j} = \begin{bmatrix} 1 & 1 & 0 & 0 & 0 & 0 & 0 & 0 \\ 0 & 0 & 1 & 1 & 0 & 0 & 0 & 0 \\ 0 & 0 & 0 & 0 & 0 & 0 & 1 & 1 \\ 1 & 1 & 2 & 2 & 1 & 1 & 1 & 1 \\ 1 & 1 & 0 & 0 & 1 & 1 & 0 & 0 \\ 0 & 0 & 2 & 2 & 1 & 1 & 0 & 0 \\ 0 & 0 & 0 & 0 & 1 & 1 & 1 & 1 \end{bmatrix}$$

The kinetic exponent matrix $n_{i,j}$ defines the exponent of species i in the rate law of reaction j. For elementary irreversible steps, the rate-law exponents coincide with the absolute stoichiometric coefficients of the reactants. This is defined by the condition:

$$n_{i,j} = \begin{cases} \nu_{i,j} & \text{if } \varepsilon_{i,j} = -1 \\ 0 & \text{otherwise} \end{cases}$$

Applying this condition to the illustrative example yields:

$$n_{i,j} = \begin{bmatrix} 1 & 0 & 0 & 0 & 0 & 0 & 0 & 0 \\ 0 & 0 & 1 & 0 & 0 & 0 & 0 & 0 \\ 0 & 0 & 0 & 0 & 0 & 0 & 1 & 0 \\ 1 & 0 & 2 & 0 & 0 & 1 & 1 & 0 \\ 0 & 1 & 0 & 0 & 1 & 0 & 0 & 0 \\ 0 & 0 & 0 & 2 & 1 & 0 & 0 & 0 \\ 0 & 0 & 0 & 0 & 0 & 1 & 0 & 1 \end{bmatrix}$$

For elementary irreversible steps, the rate-law exponents coincide with the reactant stoichiometric coefficients, and therefore $n_{i,j} = \nu_{i,j}$ for this illustrative example.

Assuming representative operating parameters $\tau = 0.1$, $A/V = 5\times10^5$, and inlet gas concentrations

$\{x_1^{in}, x_2^{in}, x_3^{in}\} = \{1, 1, 0\}$,

the gas-phase species balances become

$$\begin{aligned}
\text{A:} \quad & \frac{1 - x_1}{0.1} + 5 \times 10^5(-r_1 + r_2) = 0 \\
\text{B}_2: \quad & \frac{1 - x_2}{0.1} + 5 \times 10^5(-r_3 + r_4) = 0 \\
\text{C:} \quad & \frac{0 - x_3}{0.1} + 5 \times 10^5(-r_7 + r_8) = 0
\end{aligned}$$

The surface-species balances are

$$\begin{aligned}
\text{A*:} \quad & r_1 - r_2 - r_5 + r_6 = 0 \\
\text{B*:} \quad & 2r_3 - 2r_4 - r_5 + r_6 = 0 \\
\text{C*:} \quad & r_5 - r_6 + r_7 - r_8 = 0
\end{aligned}$$

and the site-balance constraint is

$$x_4 + x_5 + x_6 + x_7 = 1$$

The elementary reaction rates are written in the general form

$$r_j = k_j \prod_{i \in \mathcal{I}} x_i^{n_{i,j}}, \qquad j \in \mathcal{R}$$

which gives, for this reaction network,

$$\begin{aligned}
r_1 &= k_1 x_1 x_4 \\
r_2 &= k_2 x_5 \\
r_3 &= k_3 x_2 x_4^2 \\
r_4 &= k_4 x_6^2 \\
r_5 &= k_5 x_5 x_6 \\
r_6 &= k_6 x_4 x_7 \\
r_7 &= k_7 x_3 x_4 \\
r_8 &= k_8 x_7
\end{aligned}$$

The rate constants depend on the reaction type. For adsorption steps,

$$k_j = s_j \left(\frac{RT}{2\pi M_i}\right)^{1/2}, \qquad j \in \mathcal{R}_{ads}$$

where s_j is the sticking coefficient and M_i is the molecular mass of the adsorbing gas-phase species.

For surface reactions,

$$k_j = A_j T^{b_j} \exp\left(-\frac{E_{a,j}}{RT}\right) S_{den}, \qquad j \in \mathcal{R}_{sur}$$

where A_j, b_j, and $E_{a,j}$ are the modified Arrhenius parameters and S_{den} is the site-density scaling factor.

Solving the steady-state balances at each experimental temperature allows the estimation of the kinetic parameters from the observable gas-phase concentrations. Once the parameters are identified, the model can be used to compute additional kinetic descriptors such as degree of rate control, apparent activation energy, and reaction orders.

Table S1: Definitions of algorithmic parameters used in the KKT-based iterative method.

Parameter	Definition
ϵ_{feas}	Feasibility tolerance for equality-constraint residuals.
ϵ_{kkt}	Convergence tolerance for the KKT residual.
$\epsilon_{\text{restore}}$	Threshold for activating the feasibility-restoration step.
$\epsilon_{\text{pseudoinverse}}$	Singular-value cutoff used in the SVD-based pseudoinverse.
ϵ_{stag}	Tolerance used to detect stagnation in residual reduction.
α_{min}	Minimum allowable step size in the line search.
$\beta_{\text{backtrack}}$	Step-size reduction factor in backtracking line search.
c_1	Armijo sufficient-decrease parameter.
δ_{damp}	Damping factor applied to Lagrange multipliers during stagnation.
C_{stag}	Counter for consecutive stagnant iterations.
N_{stag}	Maximum allowed stagnation count before perturbation/damping.

S2 Reaction Mechanism

Table S2: Elementary reaction mechanisms for the three benchmark systems

**System 1**	**System 2**	**System 3**
R_1: $NO + \cdot \longrightarrow NO\cdot$	R_1: $H_2 + 2\cdot \longrightarrow 2H\cdot$	R_1: $H_2(g) + 2\cdot \longrightarrow 2H\cdot$
R_2: $NO\cdot \longrightarrow NO + \cdot$	R_2: $2H\cdot \longrightarrow H_2 + 2\cdot$	R_2: $2H\cdot \longrightarrow H_2(g) + 2\cdot$
R_3: $NO\cdot + \cdot \longrightarrow N\cdot + O\cdot$	R_3: $N_2 + 2\cdot \longrightarrow 2N\cdot$	R_3: $CO_2(g) + H\cdot \longrightarrow HCOO\cdot$
R_4: $N\cdot + N\cdot \longrightarrow N_2 + 2\cdot$	R_4: $2N\cdot \longrightarrow N_2 + 2\cdot$	R_4: $HCOO\cdot \longrightarrow CO_2(g) + H\cdot$
R_5: $NO\cdot + N\cdot \longrightarrow N_2O\cdot + \cdot$	R_5: $NH_3 + \cdot \longrightarrow NH_3\cdot$	R_5: $HCOO\cdot + H\cdot \longrightarrow HCOOH\cdot + \cdot$
R_6: $N_2O\cdot \longrightarrow N_2O + \cdot$	R_6: $NH_3\cdot \longrightarrow NH_3 + \cdot$	R_6: $HCOOH\cdot + \cdot \longrightarrow HCOO\cdot + H\cdot$

**System 1**	**System 2**	**System 3**
R_7: $CO + \cdot \longrightarrow CO\cdot$	R_7: $NH_3\cdot + \cdot \longrightarrow NH_2\cdot + H\cdot$	R_7: $HCOOH\cdot + H\cdot \longrightarrow H_2COOH\cdot + \cdot$
R_8: $CO\cdot \longrightarrow CO + \cdot$	R_8: $NH_2\cdot + H\cdot \longrightarrow NH_3\cdot + \cdot$	R_8: $H_2COOH\cdot + \cdot \longrightarrow HCOOH\cdot + H\cdot$
R_9: $CO\cdot + O\cdot \longrightarrow CO_2 + 2\cdot$	R_9: $NH_2\cdot + \cdot \longrightarrow NH\cdot + H\cdot$	R_9: $H_2COOH\cdot + \cdot \longrightarrow H_2CO\cdot + OH\cdot$
	R_{10}: $NH\cdot + H\cdot \longrightarrow NH_2\cdot + \cdot$	R_{10}: $H_2CO\cdot + OH\cdot \longrightarrow H_2COOH\cdot + \cdot$
	R_{11}: $NH\cdot + \cdot \longrightarrow N\cdot + H\cdot$	R_{11}: $OH\cdot + H\cdot \longrightarrow H_2O\cdot + \cdot$
	R_{12}: $N\cdot + H\cdot \longrightarrow NH\cdot + \cdot$	R_{12}: $H_2O\cdot + \cdot \longrightarrow OH\cdot + H\cdot$
		R_{13}: $H_2CO\cdot + H\cdot \longrightarrow H_3CO\cdot + \cdot$
		R_{14}: $H_3CO\cdot + \cdot \longrightarrow H_2CO\cdot + H\cdot$
		R_{15}: $H_3CO\cdot + H\cdot \longrightarrow CH_3OH\cdot + \cdot$
		R_{16}: $CH_3OH\cdot + \cdot \longrightarrow H_3CO\cdot + H\cdot$
		R_{17}: $CO_2(g) + \cdot \longrightarrow CO_2\cdot$
		R_{18}: $CO_2\cdot \longrightarrow CO_2(g) + \cdot$
		R_{19}: $CO_2\cdot + H\cdot \longrightarrow COOH\cdot + \cdot$
		R_{20}: $COOH\cdot + \cdot \longrightarrow CO_2\cdot + H\cdot$
		R_{21}: $COOH\cdot \longrightarrow CO(g) + OH\cdot$
		R_{22}: $CO(g) + OH\cdot \longrightarrow COOH\cdot$
		R_{23}: $H_2O\cdot \longrightarrow H_2O(g) + \cdot$
		R_{24}: $H_2O(g) + \cdot \longrightarrow H_2O\cdot$
		R_{25}: $CH_3OH\cdot \longrightarrow CH_3OH(g) + \cdot$
		R_{26}: $CH_3OH(g) + \cdot \longrightarrow CH_3OH\cdot$

References
